# Mapping the Evidence for Cumulative Adverse Effects of Offshore Wind Installations on Birds

**DOI:** 10.1007/s00267-026-02599-7

**Published:** 2026-08-21

**Authors:** Leon A. Green-Tkacenko, Michael C. Allen, Julie L. Lockwood

**Affiliations:** 1 Rutgers Climate and Energy Institute, Rutgers University, New Brunswick, NJ USA; 2 Map of Life Solutions, New Haven, CT USA; 3 Department of Ecology, Evolution, and Natural Resources, Rutgers University, New Brunswick, NJ USA

**Keywords:** Birds, Cumulative adverse effects, Offshore wind, Renewable energy, Systematic review, Evidence mapping

## Abstract

Offshore wind (OSW) energy is rapidly expanding worldwide as countries aim to decarbonize their electrical grids. However, as OSW scales up to meet increasing demand, concerns about cumulative environmental effects have mounted. Over the past decade, empirical and theoretical studies on cumulative effects, especially on birds, have increased apace. We conducted a systematic review of the empirical literature on birds stored in the energy impact repository *Tethys* to determine how often different categories of evidence identified as important for understanding cumulative adverse effects have been measured. We used an evidence-mapping approach, aligning measurements recorded in *Tethys* with a published typology of evidence for cumulative adverse effects. Although many frameworks for understanding cumulative effects exist, rigorous quantification of measured outcomes is lacking. Our findings show that most empirical studies have identified which bird species are vulnerable to OSW effects (46%) or provided baseline data for species prior to OSW construction (40%), both of which are fundamental to understanding cumulative effects. In contrast, only 1% examined the nature of effect pathways (additive, synergistic, countervailing), and less than 3% sought to identify acceptable mortality thresholds for species populations affected by OSW. Additionally, there is a strong geographic bias toward the North Atlantic Ocean. Our analysis highlights critical gaps in the evidence required to understand the cumulative adverse effects of OSW on birds, guiding future research toward key areas where evidence is lacking.

## Introduction

Offshore wind (OSW) energy is a rapidly growing part of global efforts to combat anthropogenic climate change and increase energy supplies (Wang et al., 2024). However, the development of OSW energy may also cause negative environmental effects, including direct mortality and habitat loss for certain bird species (Croll et al., 2022; Drewitt and Langston, 2006; Fox and Petersen, 2019; Furness et al., 2013; Watson et al., 2025). Currently, existing OSW facilities make up only a small fraction of what's planned: 68 gigawatts as of 2023, with a pipeline of 454 gigawatts (McCoy et al., 2024). As the number of OSW facilities increases, especially when they are closely spaced within regions, uncertainties remain about their cumulative impacts on bird populations (Goodale and Milman, 2016). Frameworks have been proposed to conceptualize and measure these effects (e.g., Goodale and Milman, 2016), but no study has assessed how well the current empirical literature can inform them. To fill this gap, we use an evidence mapping approach, a systematic search to identify knowledge gaps (Miake-Lye et al., 2016). We use this approach to evaluate available information that can inform a highly cited cumulative OSW effects framework (Goodale and Milman, 2016), examining how comprehensively the necessary data categories have been studied. This analysis clarifies what we already know and identifies knowledge gaps, guiding future research to better understand the combined effects of OSW on birds.

OSW installations effects on birds include direct mortality from turbine collisions (Allison et al., 2019; Hüppop et al., 2006; Hüppop et al., 2016); habitat loss, including both direct loss due to wind farm avoidance (Dierschke et al., 2016; Furness et al., 2013; Lamb et al., 2024) and indirect loss due to displacement of food sources (Allison et al., 2019); and barrier effects that disrupt migration (Fox and Petersen, 2019, Allison et al., 2019; Watson et al., 2025). Of these, direct mortality and habitat loss have received the most attention scientifically, and barrier effects—while potentially important for energetic balance in migratory species—are less well documented (Masden et al., 2010a; Masden et al., 2009). Most published studies focus on the impacts of single OSW developments (Goodale and Milman, 2016). However, as OSW facilities grow in number, there is concern that the effects of individual OSW farms may compound across facilities or interact synergistically with other anthropogenic effects, causing adverse impacts on entire bird populations (Goodale and Milman, 2016). Frameworks that define the elements necessary for assessing cumulative effects emphasize a systematic approach to evidence collection and evaluation (e.g., Croll et al., 2022; Ferguson et al., 2025; Goodale and Milman, 2016; Masden et al., 2010b; Williams et al., 2024). Each of the categories identified in cumulative adverse-effect assessments is important in its own right, but they are also interrelated. For example, if we know which species are most at risk to OSW but do not know their baseline population abundance, we cannot adequately monitor effects. Thus, missing evidence from one or more categories can leave an important hole in the knowledge base and dilute the usefulness of categories for which we do hold a comprehensive understanding.

For complex and uncertain questions, evidence mapping can help to organize existing empirical information relative to the four categories of a Rumsfeld matrix (Hald-Mortensen, 2025): things we know and understand (known knowns), things we know we do not understand (known unknowns), things we understand but have failed to recognize as important (unknown knowns), and things we do not know we do not understand (unknown unknowns). This categorization is useful for prioritizing research and reducing uncertainty related to cumulative adverse effects on bird populations. To accurately estimate the cumulative effects of OSW on birds, we must identify which essential framework components have substantial evidence (the “knowns”) and which lack data (the “unknowns”). Ultimately, this process informs research priorities, regulatory requirements, and resource allocation, ensuring that future efforts focus on gaps rather than on well-understood effects.

The cumulative adverse effects framework proposed by Goodale and Milman (2016) is well-suited to an evidence-mapping approach. Fundamentally, the framework classifies the different types of empirical information required to understand the cumulative adverse effects of OSW on wildlife. This taxonomic generality renders it well-suited to the diverse life histories of birds in marine environments. Building on prior efforts (e.g., Masden et al., 2010b) and drawing on well-cited work, the framework provides a mature baseline of categories that readily map to the existing literature. Moreover, since its publication in 2016, researchers, governments, and developers have had sufficient time to incorporate the framework's suggestions into research requirements and objectives.

Although the rapid growth in OSW is recent (Wang et al., 2024), the first installation was built in Denmark over 35 years ago (Olsen and Dyre, 1993). Europe's early adoption of OSW generated a substantial body of research on avian effects (Fox and Petersen, 2019), using diverse methodologies tailored to location, taxa, and monitoring goals (Allen and Campo, 2020; Fox et al., 2006). In 2011, to harness this expanding knowledge base, the Pacific Northwest National Laboratory launched *Tethys*, a repository compiling government, industry, and academic literature on marine energy effects (Copping et al., 2013; Whiting et al., 2019). Non-peer-reviewed documents have historically been difficult to access, and may present evidence that differs materially from that in the peer-reviewed literature (Whiting et al., 2019). Given the importance and difficulty of accessing non-peer-reviewed literature, the inclusion of government and industry reports in *Tethys* is crucial, as these documents significantly contribute to building an evidence base on the effects of wind energy on birds (Fernández-Bellon, 2020; Szostek et al., 2024). Here, we review nearly 10,000 studies in *Tethys*, mapping them to the Goodale and Milman (2016) framework, and assess the available evidence to evaluate the cumulative adverse effects of OSW on bird populations. Our objective was to identify which aspects of these effects have been well addressed in the literature and which have not, to guide future research and monitoring toward a more complete understanding of the risks.

## Methods

Our methods broadly involved acquiring all literature on OSW and birds from the *Tethys* database, retaining empirical studies that met our inclusion criteria, and mapping the measurements reported therein to the Goodale and Milman cumulative adverse effects framework.

### Data Source

*Tethys* is a comprehensive catalog of peer-reviewed and non-reviewed (“gray”) publications on the biological effects of marine energy production (Whiting et al., 2019). The database is maintained by the Pacific Northwest National Laboratory (PNNL) and undergoes annual peer review of its content (Copping et al., 2013; Whiting et al., 2019). As *Tethys* is actively curated, we did not require traditional search strings or terms used within other databases (e.g., Web of Science) to locate publications. Instead, we relied on built-in filtering functions within *Tethys*, using the Boolean criteria “Fixed Offshore Wind” or “Floating Offshore Wind”, and “Birds”. We then removed duplicates and retained publications from the resulting list according to our inclusion criteria, as described in the following section. *Tethys* historically focused on English-language documents. While the repository has expanded to include publications in other languages, particularly from Europe, content from Asia and the global south remains incomplete (Farr, *personal communication*). We translated all non-English publications into English before review using Google Translate or Apple Translate (the specific program for each document is identified in Supplementary Table 1). We accessed *Tethys* on 22 October 2024.

### Inclusion Criteria

We retained only publications focused on empirical measurements of OSW effects on birds, such as offshore population abundance or telemetry studies. As part of this, we excluded literature reviews and studies solely about mitigating effects; we included meta-analyses, data syntheses, and modeling publications that are clearly derived from field data (Supplementary Fig. 1). We excluded publications first based on the title, then on the abstract, introduction, or executive summary, and finally on a full-text review, including associated appendices if relevant. If a single study appeared multiple times in the database (e.g., first as a dissertation and later as a peer-reviewed publication), we included only one version, typically the peer-reviewed article. In some instances, *Tethys* separated appendices into entries distinct from the main document they were associated with; in these cases, each appendix and its associated main document were reviewed independently and then merged into a single entry for final analysis. Additionally, some preliminary reports are entirely restated in subsequent preliminary or final reports; in these cases, each report was reviewed independently and then merged into a single entry for final analysis (merged documents are identified in Supplementary Table 1).

### Translating the Framework

Goodale and Milman (2016) identified three main categories required to understand cumulative adverse effects: (1) *hazards*—identification of hazards, physical changes in the environment by human activities that may cause harm; (2) *vulnerable receptors*—focused on identifying which species are most vulnerable to those hazards; and (3) *exposure*—delineation of species exposure to harm, both spatially and temporally. For the *hazards* category, we included only publications that measured or discussed stressors other than the focal OSW facility (i.e., other OSW facilities or anthropogenic actions unrelated to OSW), or that included a general description of effect pathways that could generate cumulative effects. Studies in the *vulnerable receptors* category identified which species were most vulnerable to OSW effects, for example, by providing baseline population measurements or identifying thresholds for negative conservation outcomes. Studies in the *exposure* category identified the spatial and temporal characteristics of bird population abundance relative to OSW installations before and after construction, including effects throughout the installations' lifespans.

Goodale and Milman (2016) further subdivided these main categories of information into seven subtopics. We retained this schema but further subdivided some categories, resulting in 11 subcategories in total, described in Table 1. We mapped the evidence from each publication to these 11 subcategories. Briefly, under the category of *hazards*, we evaluated if a given publication identified the scope and scale of cumulative effects from other OSW facilities (subcategory “Source-OSW”), from other anthropogenic factors (“Source-non-OSW”), or if it sought to understand the general nature of cumulative adverse effect pathways for a species or group (“Effect Pathways”; see below). Under the *vulnerable receptors* category, we recognized the following three subcategories: identifying species most at risk (“Refine Receptors”), presenting pre-construction population data (“Clear Baselines”), and determining an acceptable amount of mortality (“Stating a Threshold”). For the *exposure* category, we subdivided Goodale and Milman’s “Determining the Temporal Boundaries of the Impact” topic into three subcategories of evidence: informing impacts across the lifespan of the OSW facility (“Temporal-Lifespan”); identifying future anthropogenic stressors that could contribute to cumulative adverse effects (“Temporal-Future Anthropogenic”); and identifying the life history traits of birds related to timing or phenology that dictate when adverse effects will occur (“Temporal-Life History”). Finally, also under the *exposure* category, we subdivided the topic “Determining the Spatial Boundaries of the Impact” into two subcategories: identifying spatial dynamics of bird populations in the context of OSW development (“Spatial-Population”), and identifying the optimal geographical placement of OSW developments in the context of bird conservation (“Spatial-Development Area”). Complete definitions for each of the 11 evidence subcategories are provided in Table 1 (see also Supplementary Table 2). We allowed each publication to address multiple categories and subcategories.

**Table 1:** Definitions for each of the 11 measurement categories we considered necessary to address the cumulative adverse effects of OSW, as refined from Goodale and Milman (2016)

Evidence category	Subcategory	Definition	Frequency	Proportion of Publications
**Hazards**	**Source-OSW (SHWF)**	Publications that look at more than one OSW farm as a source of cumulative adverse effects. These publications must look at the additional OSW farms cumulatively.	62	0.18
**Hazards**	**Source-non-OSW (SHO)**	Effects of anthropogenic activities other than OSW farms in the context of OSW farms. These other anthropogenic activities are fairly broad and can be anything from fossil fuel extraction to fishing to climate change.	37	0.10
**Hazards**	**Effect Pathways (EP)**	Publications that seek to identify the effect pathway of cumulative adverse effects for a given receptor. Cumulative effects can be additive, multiplicative (synergistic), or countervailing.	4	0.01
**Vulnerable receptors**	**Refine Receptors (RR)**	Refining receptors seeks to identify the species that are most vulnerable to OSW development. This can take the form of species-specific sensitivity indices, measures of flight height, or avoidance rates.	162	0.46
**Vulnerable receptors**	**Clear Baselines (CB)**	A clear baseline is a measurement that identifies the current state of a given species prior to the construction of an OSW project. This most frequently takes the form of population counts, but can also be recorded flight paths or density maps.	142	0.40
**Vulnerable receptors**	**Stating a Threshold (ST)**	Publications that determine a level of adverse effect that is acceptable for a given receptor. This category is for publications that identify the threshold of mortality that is acceptable on a species-specific basis.	10	0.03
**Exposure**	**Temporal- Lifespan (TB1)**	Publications that address effects through the temporal lifespan of an OSW project. This is not stating the lifespan of a project (which tends to be standardized to some extent by the manufacturer), but only those publications that address the effects of OSW in the construction, operations, and decommissioning stages.	20	0.06
**Exposure**	**Temporal-Future Anthropogenic (TB2)**	Publications which forecast future anthropogenic effects (in the framework, this includes past and present, but measuring past and present effects would constitute the vast majority of publications and not be particularly informative). This often takes the form of accounting for climate change, but can also be the consideration of OSW buildout scenarios into the future or other anthropogenic actions (e.g., forecasted fishing intensity).	19	0.05
**Exposure**	**Temporal-Life History (TB3)**	Life history traits of birds which effect the seasonality of adverse effects. Publications that measure, for a given bird species, population, counts, or movements through the annual cycle. These measurements can establish trends over the course of the entire year, or for a specific season of interest (e.g., breeding season).	165	0.47
**Exposure**	**Spatial- Population (SBP)**	Publications that quantify the geographic range of a population. This can be the foraging range of a breeding colony or the overall range of a species in a given geographic area. For the latter, we consider publications as mapping a population when they map a species to at least the country level for European nations or to the state/province level for other countries.	96	0.27
**Exposure**	**Spatial- Development Area (SBD)**	Publications that undertake marine spatial planning to identify areas that are suitable or unsuitable for the construction of OSW. These publications must incorporate birds into their decision-making protocols. This category does not include publications that simply map the sited bounds of planned OSW farms.	35	0.10

For each category, we provide details on the type of information required within a publication for the evidence it contains to be included in our review

As the “Effect Pathways” subcategory may not be immediately intuitive, we briefly elaborate on it here. Cumulative adverse effect pathways from OSW installations can be classified as additive, synergistic, or countervailing (Goodale and Milman, 2016). Additive effect pathways suppose that effects caused by an individual OSW facility (e.g., collision deaths, metabolic costs) are independent of those from other OSW facilities, and when combined, are the sum. Synergistic (multiplicative) effect pathways assume that effects increase disproportionately (e.g., non-linearly) with each new OSW installation and, when combined, exceed the sum of their individual effects. Countervailing pathways can occur when different cumulative adverse effects partially offset one another; for example, if displacement in one area reduces collision risk elsewhere (Goodale and Milman, 2016), the combined effect would be less than the sum. We determined if each publication investigated the nature of effect pathways.

In addition to mapping evidence to the Goodale and Milman (2016) framework, we recorded metadata. For each publication, we noted the document type (report, journal article, or other) and the study's geographic location (continent, region, and country, where possible). These two factors are important to understanding cumulative adverse effects at a global level. Different document types may provide evidence at different levels within categories, while also presenting potential barriers to access and use, with reports generally more difficult to access than journal articles. Geographic location is important, as birdlife across the world’s oceanic regions varies, and a well-rounded understanding would require knowledge of the specifics of each geographic region when planning OSW construction. For publications covering a single taxonomic order, we identified the order addressed. For multiple-order publications, we classified each publication as including marine birds (bird species that use the offshore space as habitat for at least part of their annual cycle), non-marine birds (birds that only pass through the offshore space), or both. Understanding the taxonomic groups involved is valuable, as research bias towards specific groups may lead to an incomplete understanding of the potential adverse effects of OSW on birds in general.

### Data Analysis

We computed summary statistics for the dataset, created heat maps, and global prevalence maps using R (R Core Team, 2025; Wickham, 2016). These visualizations helped assess which cumulative adverse effects and measurement categories were most frequently addressed by taxonomic group, country, and publication type. We ran a linear model in R to evaluate whether the observed distribution of publications across countries differed based on the number of installed OSW turbines.

## Results

**Fig. 1:**
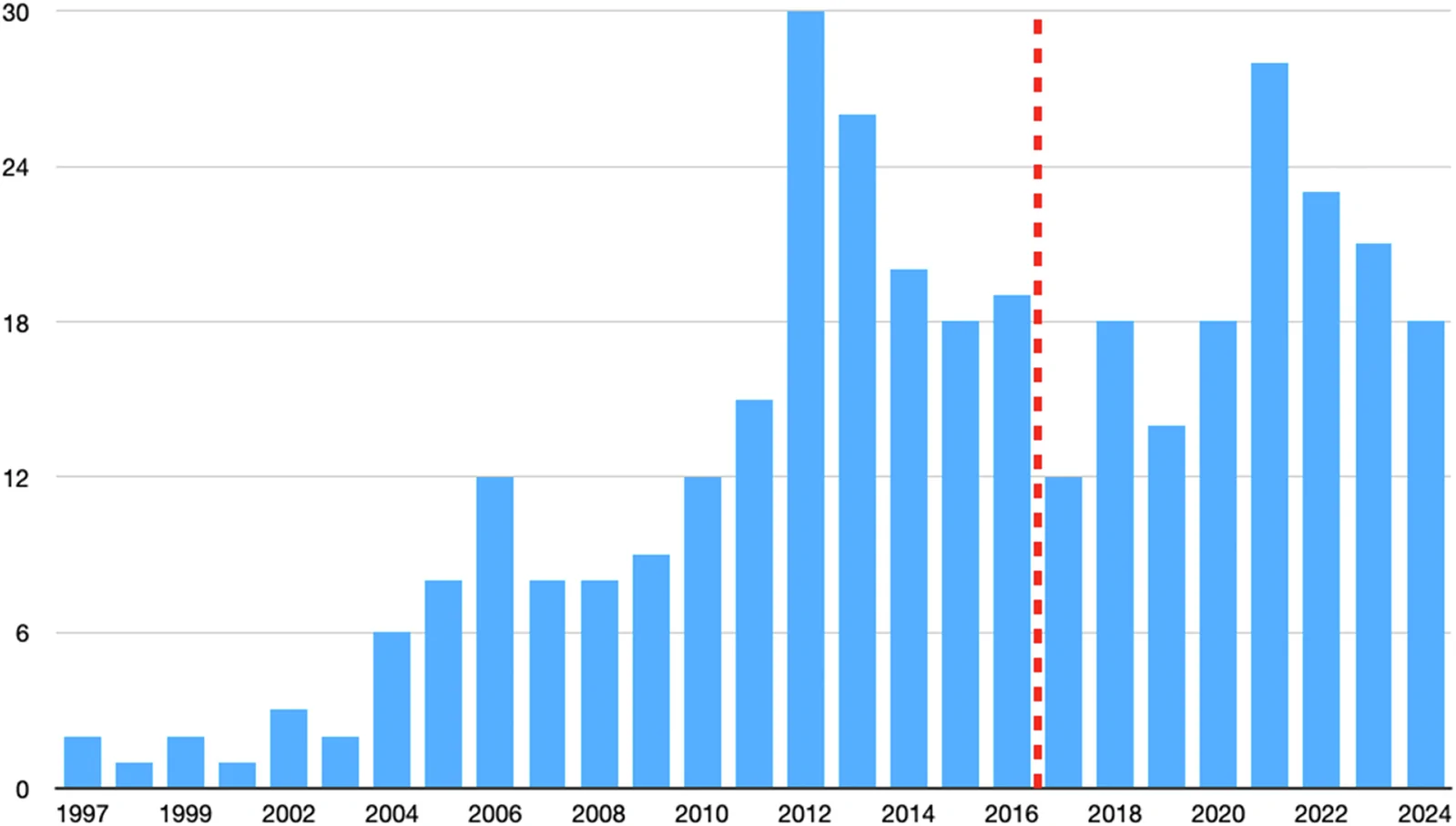
Histogram detailing the year of publication for each of the 354 publications included in our final analysis; merged preliminary reports and appendices are indexed by the year of the latest report. These publications satisfy our inclusion criteria and are present in the final dataset. The dashed red line indicates 2016, the year that the framework to which we mapped evidence was published (Goodale and Milman 2016)

Of the approximately 9920 publications found within Tethys on 22 October 2024, we identified 651 as pertinent to our review via our initial search filter. Of these 651 publications, we removed five duplicates and were unable to locate one, leaving 645 for further review. Of the 645, we excluded 269 from further consideration for failing to meet the inclusion criteria, and merged 22 appendices or intermediate reports with final report versions (see Supplementary Fig. 1). Of the 354 publications that remained, five were books or book chapters; 11 were professional presentations, papers, or posters; 117 were peer-reviewed journal articles; 208 were reports; 11 were student theses; and two were OSW working group reports. Of the included records, publication dates range from 1997 to the present (Fig. 1), with 48% published in the same year or later than the Goodale and Milman (2016) framework.

Based on these 354 publications, we found that, among the three broad cumulative adverse effect categories (hazards, vulnerable receptors, and exposure), the hazards category was addressed in 24% of publications. The vulnerable receptors and exposure categories were addressed by 69% and 64% of publications, respectively (Table 1). We found that 11% of included publications did not address any cumulative adverse effects categories. When considering the subcategories within the framework, we found that “Temporal-Life History” (TB3) was the most frequently collected measure of cumulative adverse effect on birds (165 of 354 publications, 47%), and that “Refining Receptors” (RR) was the next most commonly addressed, with 162 publications (46%). One other subcategory of evidence was represented by more than 100 publications (Table 1): measures of “Clear Baselines” (CB) with 142 (40%) publications. We found that the least frequently included measurement was “Effect Pathways” (EP), with four publications addressing this form of evidence (1%). “Stating Thresholds” (ST) and “Temporal-Future Anthropogenic” (TB2) were the next least frequently addressed, with 10 (3%) and 19 (5%) publications, respectively. The frequency with which all other measurement categories were represented in publications is shown in Table 1, indicating that they were addressed by between 20 (6%) and 96 (27%) publications.

**Fig. 2:**
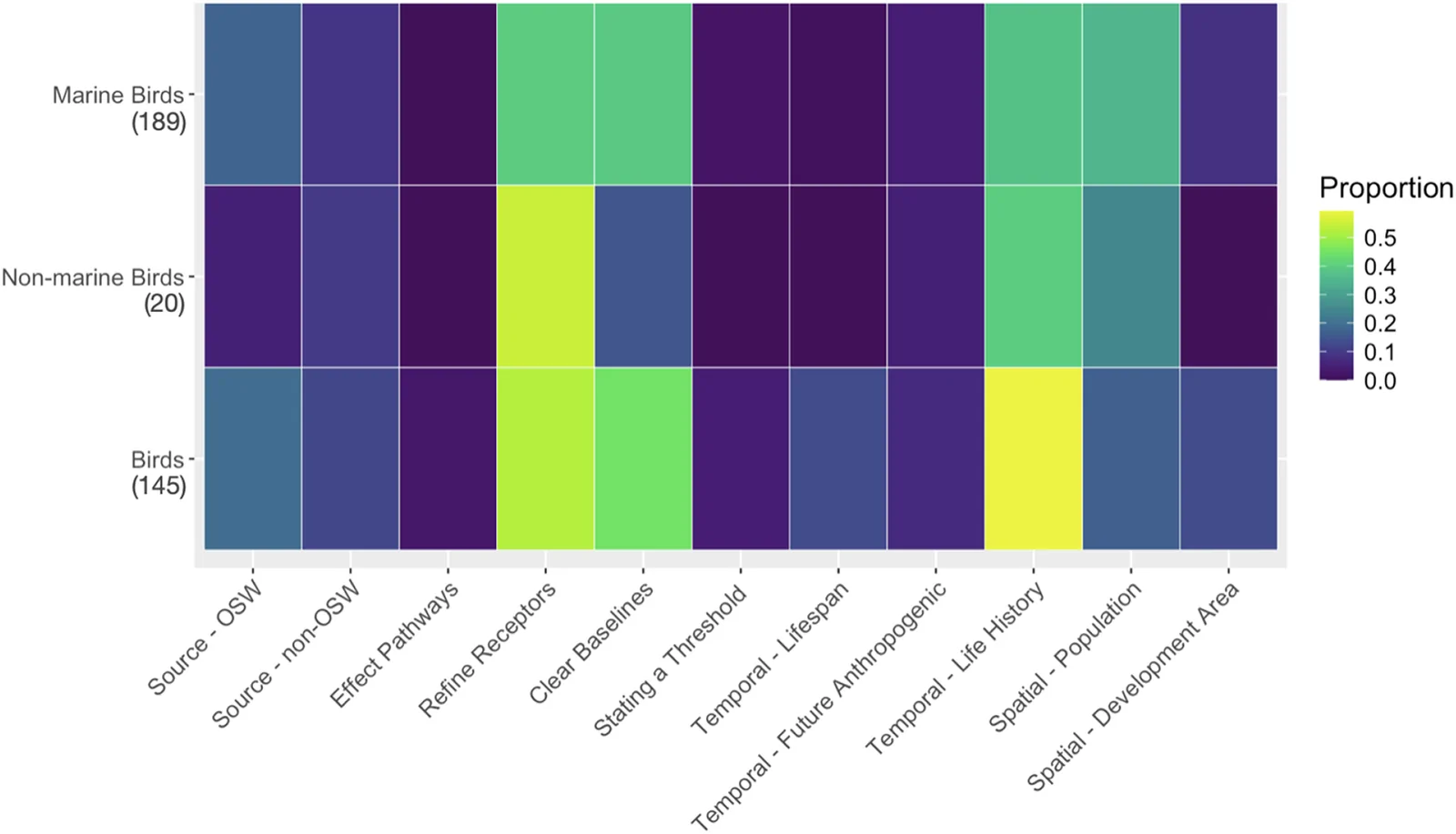
Heat map showing the proportion of publications for each taxonomic group that included measurements for each category needed to assess the cumulative adverse effects of offshore wind installations on birds. The category “Birds” refers to publications that address both marine and non-marine bird species. Lighter colors (yellow) indicate that a measurement was addressed more often in the publications we reviewed, while darker colors (blue) indicate measures that were less frequently addressed. The number of publications in each group is shown in parentheses. See Table 1 for category descriptions

We found that the frequency with which measurement categories were addressed differed between marine birds and non-marine birds. Of the 354 publications, 189 exclusively reported on marine birds, 20 exclusively on non-marine birds, and 145 on both groups (Fig. 2). We found no publications on non-marine birds that measured four effect categories: “Effect Pathway”, “Stating a Threshold”, “Temporal–Lifespan”, and “Spatial–Development Area”. For publications addressing exclusively marine birds, the only unmeasured category was “Effect Pathways”. All categories were addressed in publications examining both groups. We also found that, proportionally, four categories were more frequently addressed for non-marine birds than for marine birds: “Source-non OSW”, “Refining Receptors”, “Temporal– Future Anthropogenic”, and “Temporal–Life History”. “Source-OSW” had the largest difference between the taxonomic groups, with 17% of marine bird publications addressing it and only 5% of non-marine bird publications; a more than 3-fold difference. *“*Clear Baselines” neared the same level of difference, with 39% and 15% respectively for marine birds and non-marine birds.

We found that 82 publications in the dataset focused on a single taxonomic order of marine birds. These publications spanned five orders: Charadriiformes (34 publications), Anseriformes (26), Suliformes (13), Gaviiformes (8), and Procellariiformes (1). We found that, for the four taxonomic orders with more than one publication, there were no publications addressing “Effect Pathways” or Temporal–“Lifespan”. “Spatial- Population” was the most frequently addressed category for Charadriiformes, followed by “Clear Baselines” and “Temporal-Life History” (tie). “Clear Baselines” and “Refining Receptors” were the most frequently addressed categories for Anseriformes and Suliformes, respectively. “Source-OSW” and “Spatial-Population” were addressed more frequently amongst the subset of single-order publications than for seabirds at large, with 25% and 43% of publications compared to 17% and 35% respectively (see Supplementary Fig. 2 for comparison to Fig. 2).

**Fig. 3:**
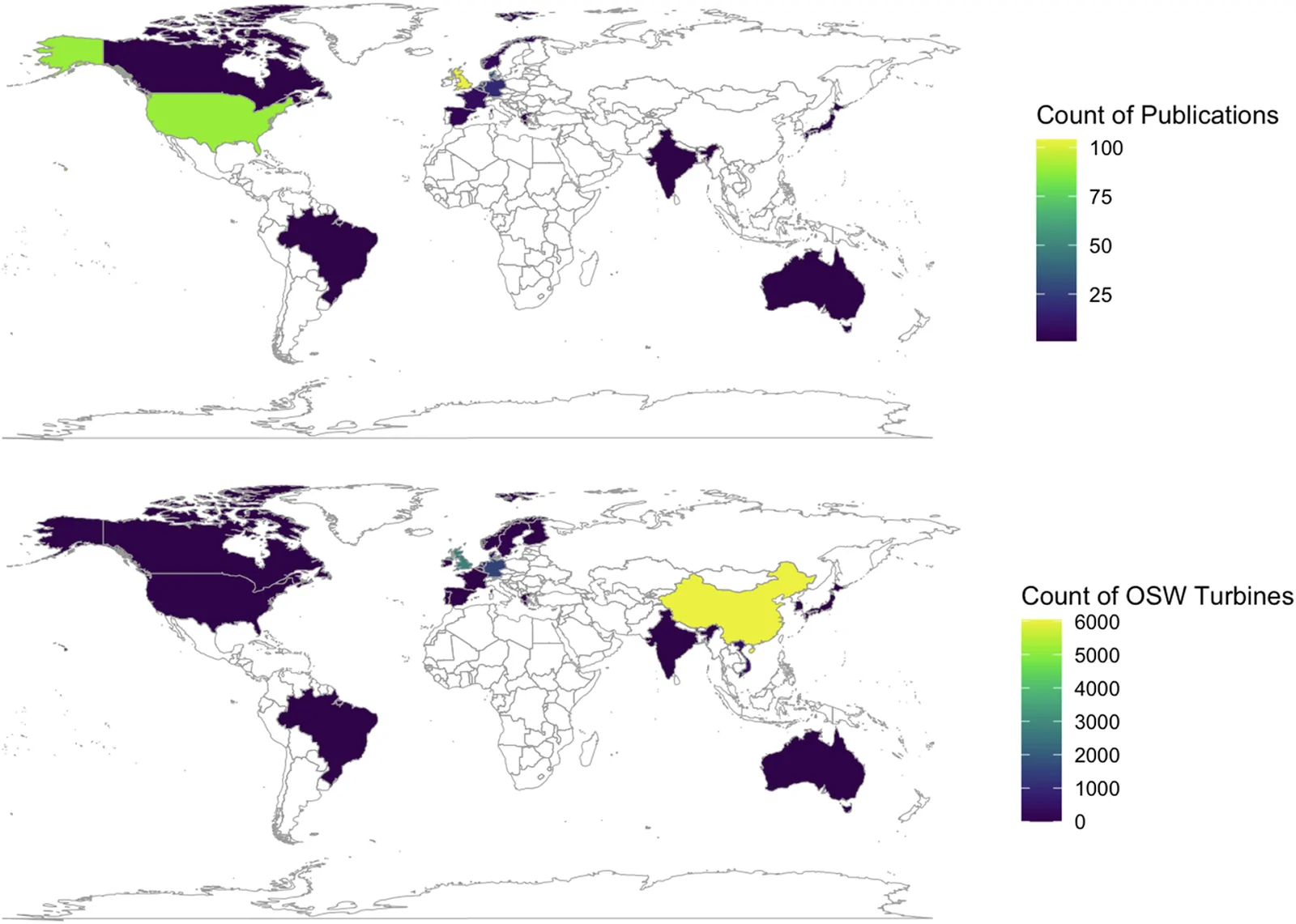
Country-level heat maps showing the geographic distribution of the 305 country-specific publications that qualified for inclusion in our dataset (top) and the number of installed OSW turbines (bottom). Darker colors indicate higher numbers of publications or turbines from that country, while white indicates no publications or OSW turbines, respectively

**Fig. 4:**
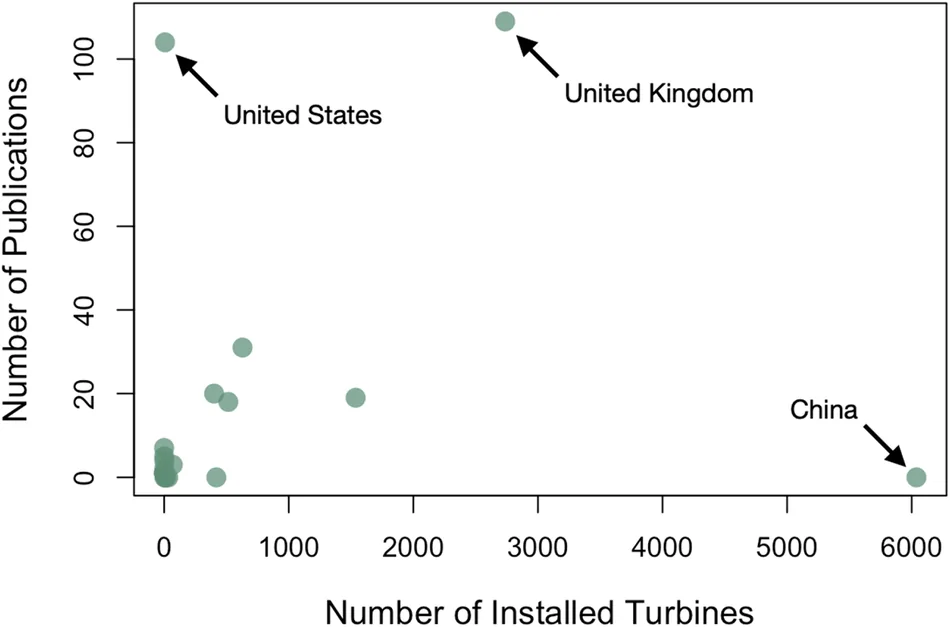
Plot showing, for each country, the number of publications included in this review compared to the number of installed offshore wind turbines. The United States, the United Kingdom, and China are labeled as potential outliers

We found that the geographic distribution of publications qualifying for inclusion in our review was highly skewed toward Europe, with 250 publications (71%). This was followed by North America, with 92, and Australasia, with four. This geographic distribution aligns with *Tethys*'s general bias towards publications in English and other European languages. There were 49 publications where we could not assign the measurements they contained to a single country. For those where we could (305), the United Kingdom had the greatest number at 104, followed by the United States at 89 (Fig. 3). Four other countries had ten or more publications qualifying for inclusion: Belgium, Denmark, Germany, and the Netherlands. Comparing the number of publications to the number of turbines in each country revealed that the United States and the United Kingdom were particularly well represented, whereas China and Vietnam were notably underrepresented among publications deposited in *Tethys* (Fig. 4).

**Fig. 5:**
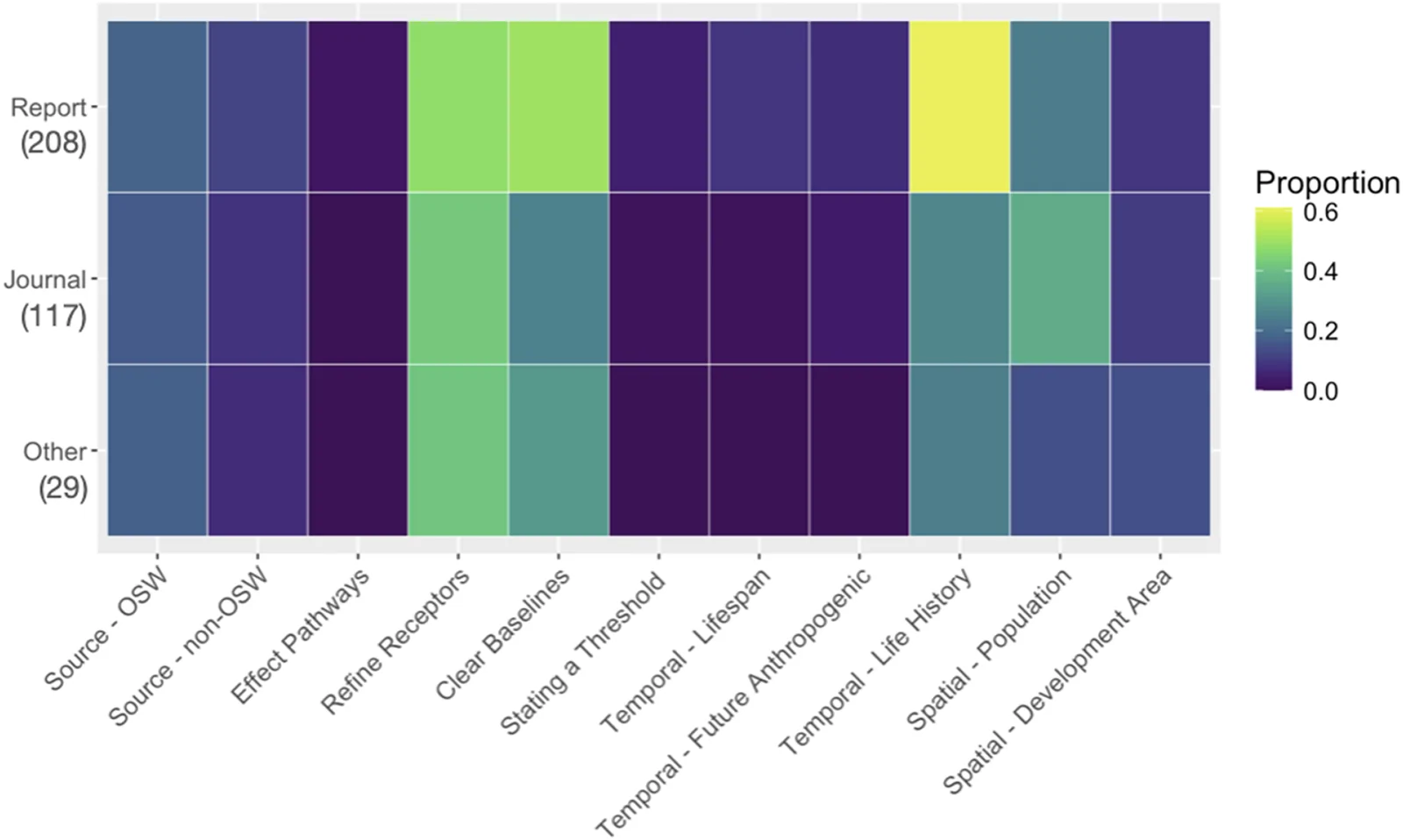
Heat map showing the proportion of *Tethys* publications for each publication type that included measurements for each category needed to understand the cumulative adverse effects of offshore wind installations on birds. Lighter colors (yellow) indicate a measurement was addressed more often within the publications reviewed, while darker colors (blue) show measures that were less frequently addressed. The number of publications in each group is noted in parentheses. See Table 1 for category descriptions

Some measurement categories were not evenly represented among peer-reviewed journal articles and reports (Fig. 5). Measures for “Temporal– Lifespan” were by far the most disproportionately addressed, with reports containing these measurements 9% of the time compared to 1% for journal articles. Measures representing “Clear Baselines” were also disproportionately included, appearing in reports nearly twice as often (50%) as in peer-reviewed articles (25%). We found that measures of “Temporal– Future Anthropogenic” and “Temporal–Life History” were included in reports twice as frequently as in peer-reviewed articles (7% and 6% for reports vs 3% and 3% for journal articles, respectively). Measures of “Spatial–Population”, on the other hand, were addressed in peer-reviewed articles (36%) far more frequently than in reports (24%). The “Effect Pathways” category was only represented in reports.

## Discussion

Understanding the cumulative adverse effects of OSW installations on birds is crucial given the global growth in development worldwide (Lees et al., 2022; Wang et al., 2024). Addressing gaps in our understanding of cumulative adverse effects can help prevent the discovery of unexpected biological effects, which could cause unnecessary delays in the transition away from fossil fuels or in meeting growing energy demands. We show that the evidence base developed over the past three decades is highly uneven across various lines of evidence required to inform cumulative adverse effects, leaving some as “known knowns” and others as “known unknowns”. Among the three broad categories of evidence described in the Goodale and Milman (2016) framework, hazards—consideration of the physical changes from anthropogenic actions by which cumulative adverse effects could manifest—was by far the least frequently addressed in birds. As OSW installations continue to expand, our lack of understanding of cumulative adverse effects could lead to unwise decisions that worsen the plight of vulnerable species. At the same time, under the precautionary principle (Searle et al., 2025), development of this important resource could also be unnecessarily slowed or restricted where knowledge of cumulative impacts is uncertain. While uncertainty is inevitable, filling gaps as we identify here will allow for greater clarity, benefiting vulnerable species and ecosystems as well as the clean energy transition.

Perhaps the most pressing “known unknown” subcategory among the lines of evidence we evaluated relates to “Effect Pathways”. This subcategory describes whether mortalities caused by bird encounters with OSW facilities are additive (effects are the sum of installed OSW farms), synergistic (effects are more than the sum of installed OSW farms), or countervailing (effects are less than the sum of installed OSW farms). We found four publications that addressed “Effect Pathways” with more than just a brief mention. However, the depth of analysis varied, with few offering detailed examinations of the likely effect pathways for several mechanisms of OSW impact on birds (e.g., Boon et al., 2010). Several other publications assumed an additive-effect pathway (e.g., Piet et al., 2021), but few provided evidence to justify their choice. At least one acknowledged the lack of evidence supporting their decision (Piet et al., 2021). While we found that additive mortality is the default assumption for effect pathways, if the actual effect pathway is multiplicative or countervailing, then any estimate of cumulative adverse effects for a population would be under- or over-estimated, respectively (Goodale and Milman, 2016). Within a single OSW farm, effect pathways between different impact categories are often considered features in collision risk models. For example, these models expressly account for the fact that birds that avoid OSW farms are less susceptible to collision (Brabant et al., 2015). Expanding this nuanced understanding to interactions among multiple installations and other anthropogenic stressors would enhance our understanding of cumulative adverse effects.

Defining thresholds beyond which adverse effects on birds lead to population declines is another urgent “known unknown”. This subcategory, under the broader category of vulnerable receptors, allows for the clear identification of the point at which additional mortality from OSW or other sources would cause cumulative negative population-level effects if exceeded (Goodale and Milman, 2016), and consequently, the level of mortality from OSW facilities that must be avoided. Our review shows that most of the current evidence base does not address this issue. Although this task is challenging due to the need for detailed knowledge of species' life histories and population status, existing methods have been applied in this area (e.g., Leopold et al., 2015; Skov et al., 2012).

The most notable method used for threshold setting is the relatively well-established practice of using potential biological removal, a method developed for marine mammals (Wade, 1998) and applied to marine birds (Dillingham and Fletcher, 2011; Richard and Abraham, 2013). Potential biological removal works much like fisheries models in that a maximum sustainable “yield” (i.e., inadvertent mortality) is estimated (Wade, 1998). This threshold-setting approach has been used infrequently in the context of OSW for marine birds (e.g., Leopold et al., 2015; Skov et al., 2012), but it has potential for wider use. We suggest that, for many species and locales, potential biological removal could be estimated given a clear understanding of baseline population information, a subcategory that is reasonably well addressed in the published literature. Nevertheless, for most species, such estimates will be highly uncertain due to a lack of detailed life-history information (Green et al., 2016). Other modeling approaches, such as individual-based models (e.g., Warwick-Evans et al., 2018) and population viability analysis (Cook and Robinson, 2017), can also enrich our understanding of potential population responses to OSW build-out scenarios. Uncertainty, however, will always remain when determining acceptable levels of mortality, and planning using the precautionary principle will remain essential into the future, with additional data helping to limit the degree of precaution needed.

We found more evidence on other subcategories within the vulnerable receptors and exposure groups—identifying species that are most at risk, based on their status before construction, geographic occurrence, and annual cycle—constituting our “known knowns”. Given this relatively robust evidence base, we suggest that a clear next step for these well-documented effects is to estimate effect sizes and identify pertinent factors influencing them via formal meta-analysis (e.g., Lamb et al., 2024). Meta-analysis cannot replace direct measurement of location-specific characteristics, and continued changes and development of OSW technology may mean that impact responses themselves change over time. Still, meta-analysis and other evidence synthesis approaches can help characterize the relative magnitude of species responses to OSW and guide site-specific sampling toward the species and effects that are most important (Lamb et al., 2024). Even as the sited locations and turbine design continue to change, information from such approaches may eventually serve to alleviate the need to direct resources towards studying species with responses that are already well known.

We found that the evidence base regarding cumulative adverse effects on marine birds is much stronger than that for non-marine birds. Understanding the effects of OSW facilities on marine birds has long been considered essential (Furness and Wade, 2012; Garthe and Hüppop, 2004), as they rely on ocean habitats for foraging, dispersal, and resting. However, many non-marine bird species may be at risk from OSW facilities during their annual over-ocean migrations (e.g., Green-Tkacenko et al., 2025; Hüppop et al., 2016; Reid et al., 2023). We observed that measurements of the cumulative effects of multiple anthropogenic sources (e.g., other wind farms) and of baseline population status prior to construction were rarely included in existing *Tethys* publications for non-marine birds. Moreover, we found that evidence supporting thresholds beyond which populations decline, and the setting of temporal and spatial limits for the OSW facilities themselves, was completely absent for non-marine birds. We acknowledge that some evidence on non-marine birds might have been missed if they were included in the broader “birds” publications, which address both non-marine birds and marine birds. Although the taxonomic focus of these publications varies, most of the species discussed are marine birds. The lack of measured evidence on the cumulative adverse effects of OSW on non-marine birds could result from a bias towards breeding-season research (Marra et al., 2015), while their aerohabitat above the ocean is less well studied than that of marine birds (e.g., Hüppop et al., 2016). In addition, small birds, such as passerines, that migrate over the ocean are often too small to be readily tracked with high-accuracy, high-frequency geolocation devices such as GPS tags (Courbis et al., 2023). Instead, our understanding of migration over water in many non-marine birds requires methodologies such as light-level geolocators, radar, and Motus tags, which do not provide the same level of spatial or (for radar) taxonomic resolution (Courbis et al., 2023). Furthermore, understanding changes in non-marine migratory birds can be complicated by a lack of baseline information due to limited available methods. Robust impact studies, such as before-after-control-impact studies, often require data that are not available even for many well-studied seabirds (Watson et al., 2025).

We found a clear bias in *Tethys* publications toward evidence from Western Europe and Eastern North America. This bias is expected due to the way the Tethys database has been constructed over time, with the database originally indexing only English-language publications. Tethys has expanded to include publications from many European nations, as reflected in our dataset; however, literature from Asia remains underrepresented. Less than 10% of the reviewed publications considered bodies of water other than the Atlantic Ocean and its associated seas, mainly the eastern Pacific and the Great Lakes. No publications provided evidence of cumulative adverse effects on birds in China's offshore waters, which currently hosts the largest number of OSW turbines (Wang et al., 2024). Additionally, there were no publications from Vietnam, another country experiencing rapid OSW development (Wang et al., 2024). This lack of information from China and Vietnam confirms that industry reports from these countries are rarely, if ever, deposited in *Tethys*, even when produced. Nonetheless, addressing and correcting this evidence bias is essential to gaining a clear understanding of the cumulative adverse effects of OSW on birds in Asia and globally. Because of global biogeographical patterns, this spatial bias also translates into a taxonomic bias. For instance, petrels, shearwaters, and albatrosses (Order Procellariformes) frequently utilize offshore ocean habitats and have significantly higher species richness in the Pacific Ocean compared to the Atlantic Ocean (Chown et al., 1998). Moreover, the East Asian-Australasian flyway is the most diverse and threatened migratory bird corridor in the world (Yong et al., 2015), and is home to critically endangered migratory species, such as the Spoon-billed Sandpiper (*Calidris pygmaea*; Birdlife International, 2021). Therefore, because the evidence of cumulative adverse effects has so far been documented only in a few locations within *Tethys*, the database contains limited recorded evidence of effects on these other species groups. This evidence gap is especially noteworthy given the recent rapid expansion of OSW across the Pacific (McCoy et al., 2024; Wang et al., 2024).

We found that both peer-reviewed and unpublished reports commonly gathered evidence supporting categories of cumulative adverse effects, though with a few notable differences. Reports are much more likely than peer-reviewed articles to include a clear baseline population status for target bird species. This outcome is likely because regulatory agencies typically require baseline field data on species abundance and distribution before approving an OSW project (e.g., European Commission, 2020). Similarly, discussions of the time frame over which species are exposed to the effects of OSW activity are more than twice as common in reports as in peer-reviewed articles. Conversely, peer-reviewed articles more frequently address the spatial boundaries of both the population and the development area than reports do. We believe these differences stem from the different spatial scales at which species are considered and mapped in reports versus peer-reviewed research. Many reports focus on mapping species at the scale of a single OSW facility and its buffer zone. In contrast, peer-reviewed studies often examine multiple facilities or broader spatial scales, sometimes covering entire countries' exclusive economic zones. In general, this highlights the importance of considering both “gray” literature and peer-reviewed studies when evaluating the breadth of evidence for cumulative adverse impacts.

Viewing the continued rapid expansion of OSW globally through the “cumulative lens” will be fundamental to fully understanding the environmental trade-offs involved across regions of the world. To that end, we encourage increased efforts to gather both new and existing evidence across all categories and subcategories identified as important for understanding OSW's cumulative adverse effects on birds. Such a “build-out” of the evidence base would not eliminate uncertainty, but it would lead to a richer understanding of potential impacts beyond the local project level and bring clarity to highly uncertain regional management decisions. Birds face risks worldwide, with nearly half of all species experiencing population declines, and climate change presents a significant future threat (Lees et al., 2022). Because of these risks, reducing carbon emissions is a top priority, but changes to land and seascapes necessary to meet this challenge will also create new issues (Lees et al., 2022). Evidence syntheses, such as this one, highlight a pathway to direct resources towards taxa and risks that are currently poorly understood. In particular, our results suggest that incorporating gray and peer-reviewed literature from poorly represented countries into accessible databases would be especially beneficial. Building a more balanced evidence base on the impact of offshore wind on birds will support efforts to decarbonize the electrical grid while also reducing overall adverse effects on biodiversity.

## Supplementary information

Supplementary Figure 1

Supplementary Figure 2

Supplementary Table 1

Supplementary Table 2

## Data Availability

No datasets were generated or analysed during the current study.
