# Supplementary material for: Mapping the Evidence for Cumulative Adverse Effects of Offshore Wind Installations on Birds
Source: Environ Manage. 2026 Aug 21;76(9):283. doi: 10.1007/s00267-026-02599-7 (PMC13498612; doi:10.1007/s00267-026-02599-7)
Supplement: Supplementary file 1 [file 267_2026_2599_MOESM1_ESM.pdf]

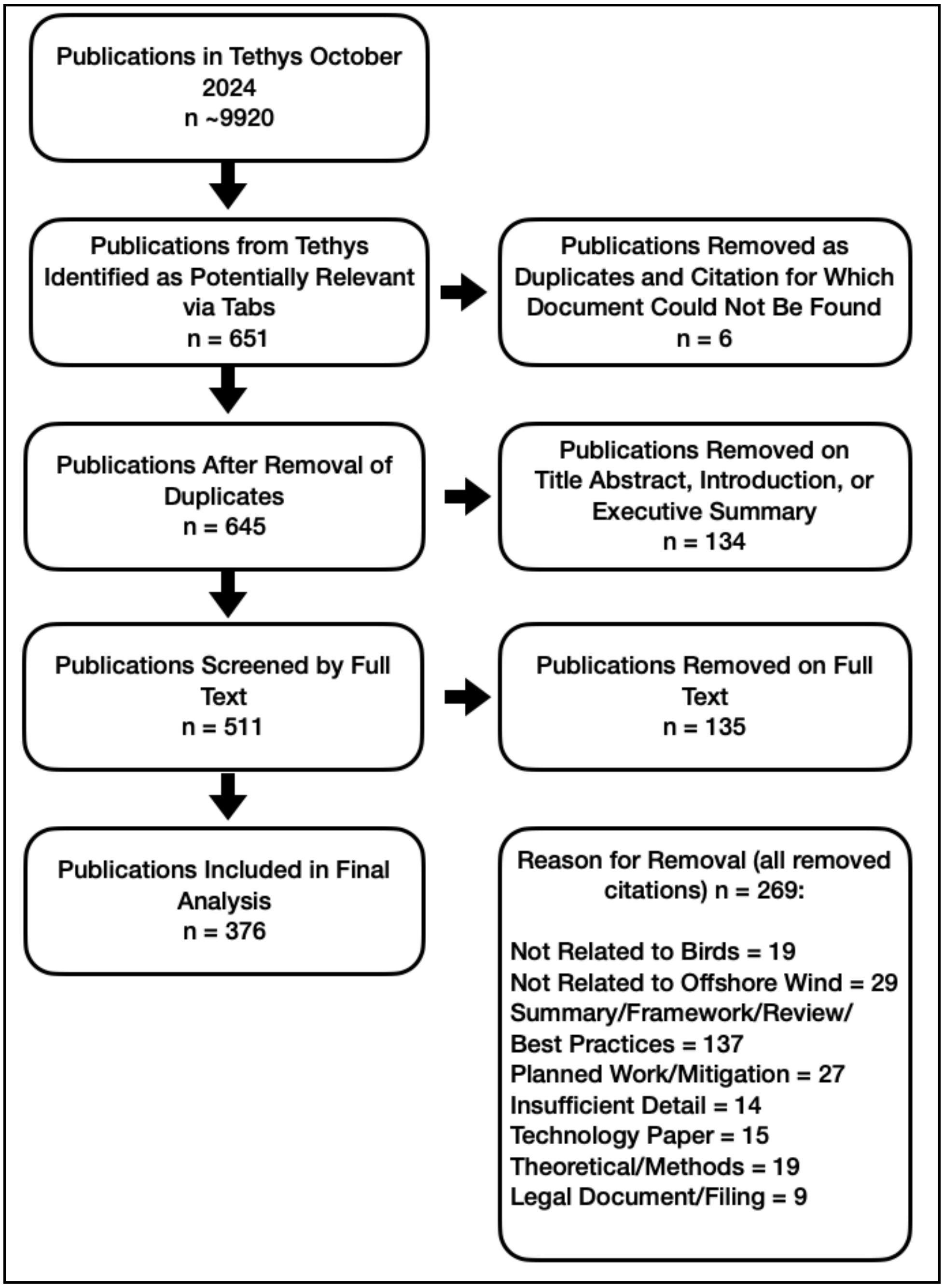

Supplementary Figure 1. Flow figure depicting how our initial publication list was refined to include only those relevant to our review goals. We include the reason for exclusion of publications based on our reading of the abstract, introduction, executive summary, and full text. Note that excluded publications could have been excludable under more than one criteria, but we show only one criteria for each here.
