# Supplementary material for: Mapping the Evidence for Cumulative Adverse Effects of Offshore Wind Installations on Birds
Source: Environ Manage. 2026 Aug 21;76(9):283. doi: 10.1007/s00267-026-02599-7 (PMC13498612; doi:10.1007/s00267-026-02599-7)
Supplement: Supplementary file 2 [file 267_2026_2599_MOESM2_ESM.pdf]

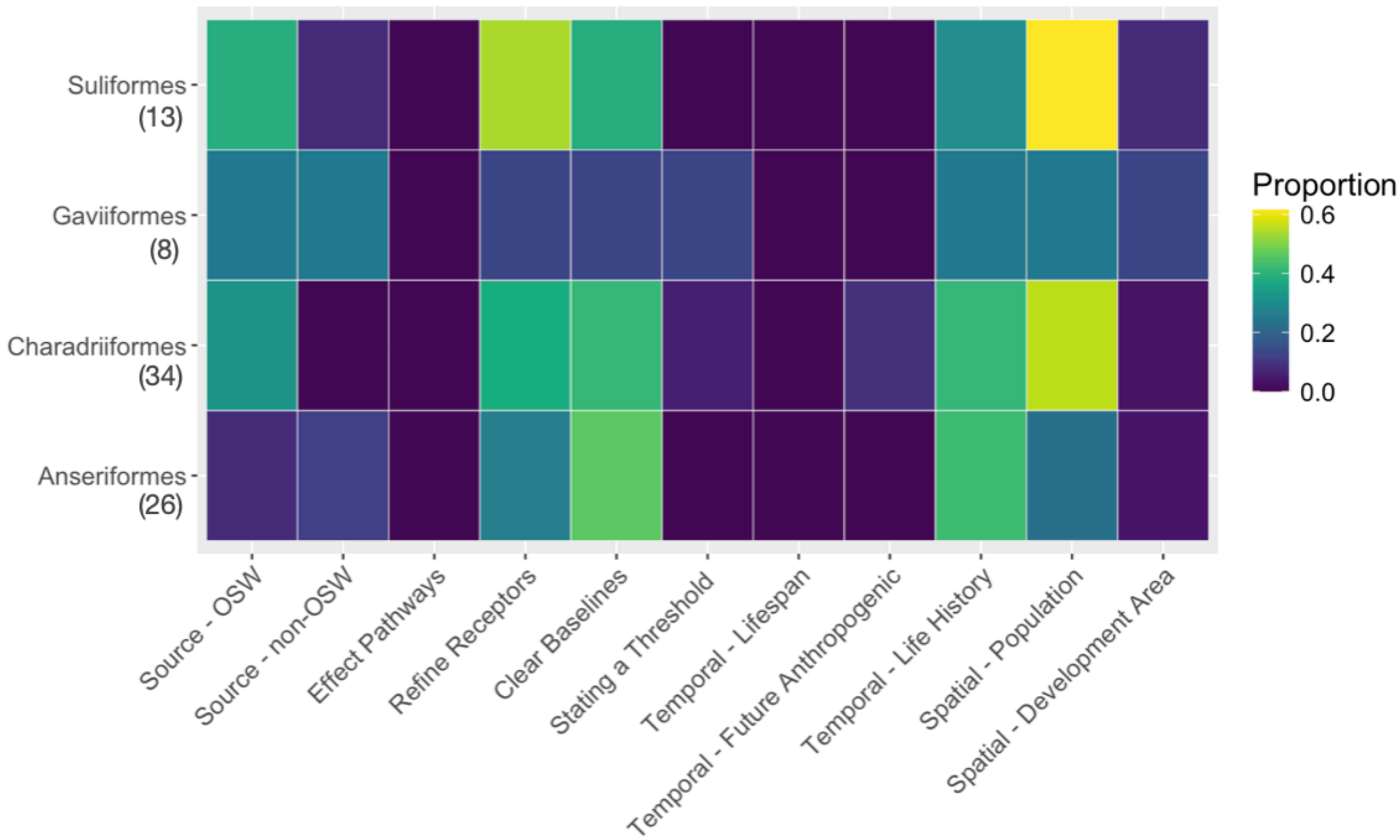

Supplementary Figure 2. Heat map showing the proportion of publications specific to each taxonomic order of marine waterbirds that included measurements for each category needed to assess the cumulative adverse effects of offshore wind installations on birds. Lighter colors (yellow) indicate that a measurement was addressed more often in the publications we reviewed, while darker colors indicate measures that were less frequently addressed. The number of publications in each group is shown in parentheses.
