# Supplementary material for: Mapping the Evidence for Cumulative Adverse Effects of Offshore Wind Installations on Birds
Source: Environ Manage. 2026 Aug 21;76(9):283. doi: 10.1007/s00267-026-02599-7 (PMC13498612; doi:10.1007/s00267-026-02599-7)
Supplement: Supplementary file 3 [file 267_2026_2599_MOESM3_ESM.pdf]

Supplementary Table 1. References for all publications meeting the defined inclusion criteria. * - Translated by Google Translate, # - Translated by Apple Translate

~ - Merged as Appendix, & - Merged as Preliminary/Final Reports

1	Adams, J., E. Kelsey, J. Felis, and D. Pereksta. 2016. Collision and Displacement Vulnerability Among Marine Birds of the California Current System Associated with Offshore Wind Energy Infrastructure. US Geological Survey (USGS).
2	Ainley, D. G., E. PORZIG, D. ZAJANC, and L. B. SPEAR. 2015. Seabird flight behavior and height in response to altered wind strength and direction. Marine Ornithology 43:25-36.
3	Allison, T., S. Perkins, and M. Perry. 2009. Determining Night-Time Distribution of Long-Tailed Ducks Using Satellite Telemetry. Audubon Society.
4	Arundale, M., A. Goulding, K. Rogerson, K. St John Glew, S. Warford, and L. Jervis. 2021. Digital Aerial Wildlife Survey of BOEM Lease Area OCS-A 520 (Beacon Wind) Annual Report: December 2019 to November 2020. Normandeau Associates Inc.
5	Arundale, M., S. Warford, and J. Willmott. 2022. Year 2 Digital Aerial Wildlife Survey of BOEM Lease Area OCS-A 520 Quarterly Report: July 2021 to October 2021. Normandeau Associates Inc.
6	Arundale, M., S. Warford, J. Willmott, S. Bloor, B. Goddard, and L. Jervis. 2021. Year 2 Digital Aerial Wildlife Survey of BOEM Lease Area OCS-A 520 Monthly Report (Year 2): May II 2021. Normandeau Associates Inc.
7	Arundale, M., S. Warford, J. Willmott, S. Bloor, B. Goddard, and L. Jervis. 2021. Year 2 Digital Aerial Wildlife Survey of BOEM Lease Area OCS-A 520 Monthly Report (Year 2): May I 2021. Normandeau Associates Inc.
8	Arundale, M., A. Williams, S. Warford, J. Robinson Willmott, and L. Jervis. 2021. Year 2 Digital Aerial Wildlife Survey of BOEM Lease Area OCS-A 0512: Combined Monthly Survey Report (Year 2): February 2019-December 2019. Normandeau Associates Inc.
~9	Atlantic Shores Offshore Wind, L. L. C. 2023. Atlantic Shores Offshore Wind Construction and Operations Plan for Commercial Lease OCS. Atlantic Shores Offshore Wind, LLC, New Jersey, USA.
*10	Aumüller, R., K. Boos, S. Freienstein, K. Hill, and R. Hill. 2013. Do migrating birds avoid offshore wind turbines? A method to investigate and analyze reactions of diurnally migrating birds to offshore wind farms. Vogelwarte 51:3-13.
11	Baisner, J. A., L. J. Andersen, A. Findsen, Y. W. S. Granath, Ø. K. Madsen, and M. Desholm. 2010. Minimizing Collision Risk Between Migrating Raptors and Marine Wind Farms: Development of a Spatial Planning Tool. Environmental Management 46:801-808.
12	Bald, J., J. Franco, I. Menchaca, Y. Torre-Enciso, and D. Marina. 2017. Impact on seabirds of new offshore wind energy test and demonstration projects in the Biscay Marine Energy Platform (BiMEP, N. Spain). Page 1 in 3rd Bilbao Marine Energy Week 2017.
13	Barton, C., and C. Pollock. 2004. Review of the Potential Impacts of Offshore Wind Farms on Migrating and Overwintering Swans and Geese in the SEA 5 Area. Cork Ecology.

**14**	Bates, E., K. Gianou, J. Hennessey, K. Lassiter, A. McCord, C. Niles, J. Doerpinghaus, M. Culver, and L. Whiting. 2017. Marine Spatial Plan for Washington's Pacific Coast. Washington State Department of Ecology.
**15**	Bemmelen, V. A. S. R., J. J. Leemans, P. M. Collier, W. M. R. Green, P. R. Middelveld, B. C. Thaxter, and C. R. Fijn. 2024. Avoidance of offshore wind farms by Sandwich Terns increases with turbine density. Ornithological Applications 126.
**#16**	Benich, U. 2021. Estudio de la viabilidad económica y energética de la construcción de un parque eólico offshore en el litoral español. Undergrad Capstone. Universitat Politècnica de Catalunya (UPC).
**17**	Benjamins, S., E. Masden, and M. Collu. 2020. Integrating Wind Turbines and Fish Farms: An Evaluation of Potential Risks to Marine and Coastal Bird Species. Journal of Marine Science and Engineering 8:414.
**18**	Best, D. B., and N. P. Halpin. 2019. Minimizing wildlife impacts for offshore wind energy development: Winning tradeoffs for seabirds in space and cetaceans in time. PLOS ONE 14:e0215722.
**~19**	Biodiversity Research, I. 2020. Revolution Wind COP Appendix AA: Assessment of the Potential Effects of the Revolution Wind Offshore Wind Farm on Birds and Bats. Biodiversity Research Institute, Providence, RI, USA.
**~20**	Biodiversity Research, I. 2021. Atlantic Shores Offshore Wind COP Appendix II-F3: Red Knot Tagging Study Memorandum. Atlantic Shores Offshore Wind, LLC.
**~21**	Biodiversity Research, I. 2022. Kitty Hawk North Wind COP Appendix U: Assessment of the Potential Effects of the Kitty Hawk Offshore Wind Project on Bats and Birds. Avangrid Renewables.
***22**	Biotope. 2020. Parc éolien en mer de Saint-Nazaire – Suivi de l'avifaune et de la mégafaune marine. Parc éolien en mer de Saint-Nazaire.
**23**	Blew, J., M. Hoffmann, G. Nehls, and V. Hennig. 2008. Investigations of the Bird Collision Risk and the Responses of Harbour Porpoises in the Offshore Wind Farms Horns Rev, North Sea, and Nysted, Baltic Sea, in Denmark - Part I: Birds. BioConsult SH.
**24**	Bloor, P., and A. Wratten. 2006. Aerial Surveys of Waterbirds in Strategic Wind Farm Areas. UK Department of Trade and Industry (DTI).
**25**	(BOEM), B. o. O. E. M., U. F. a. W. S. (USFWS), U. A. C. o. Engineers, U. C. Guard, and E. V. Rancheria 2024. Commercial Wind Lease Issuance on the Pacific Outer Continental Shelf, Offshore Oregon Final Environmental Assessment.
**26**	Boezaart, A., J. Edmonson, C. Standridge, N. Pervez, N. Desai, B. Williams, A. Clark, D. Zeitler, S. Kendall, B. Biddanda, A. Steinman, B. Klatt, J. Gehring, K. Walter, and E. Nordman. 2014. Lake Michigan Offshore Wind Feasibility Assessment. Grand Valley State University.
**27**	Bogdanova, M., L. Buckingham, M. Newell, S. Wanless, M. Harris, C. Andrews, T. Morley, E. Weston, R. Swann, M. Canham, S. Bennett, N. Cox, M. Quintin, and F. Daunt. 2021. Hywind Scotland Ornithological Monitoring Programme: Overwintering distributions of common guillemot and razorbill populations in Eastern Scotland. UK Centre for Ecology and Hydrology.

**28**	Bonthu, S., R. Purvaja, S. K. Singh, D. Ganguly, R. Muruganandam, T. Paul, and R. Ramesh. 2024. Offshore wind energy potential along the Indian Coast considering ecological safeguards. Ocean & Coastal Management 249:107017.
**29**	Boon, A., R. ter Hofstede, C. Klok, M. Leopold, G. Blacquière, M. Poot, R. Kastelein, and C. Camphuysen. 2010. Monitoring and Researching Ecological Effects of Dutch Offshore Wind Farms. Deltares (NL).
**30**	Borkenhagen, K., A.-M. Corman, and S. Garthe. 2018. Estimating flight heights of seabirds using optical rangefinders and GPS data loggers: a methodological comparison. Marine Biology 165.
**31**	Brabant, R., N. Vanermen, M. W. E. Stienen, and S. Degraer. 2015. Towards a cumulative collision risk assessment of local and migrating birds in North Sea offshore wind farms. Hydrobiologia 756:63-74.
**32**	Bradaric, M. 2022. On the radar: Weather, bird migration and aeroconservation over the North Sea. PhD Thesis. University of Amsterdam.
**33**	Bradarić, M., B. Kranstauber, W. Bouten, V. H. Gasteren, and S. J. Baranes. 2024. Drivers of flight altitude during nocturnal bird migration over the North Sea and implications for offshore wind energy. Conservation Science and Practice 6.
**34**	Bradarić, M., B. Kranstauber, W. Bouten, and J. Shamoun-Baranes. 2024. Forecasting nocturnal bird migration for dynamic aeroconservation: The value of short-term datasets. Journal of Applied Ecology 61:1147-1158.
**35**	Bradbury, G., M. Trinder, B. Furness, N. A. Banks, G. W. R. Caldow, and D. Hume. 2014. Mapping Seabird Sensitivity to Offshore Wind Farms. PLOS ONE 9:e106366.
**36**	Brady, D. 2015. Environmental Monitoring Report for VolturnUS Deployment in Castine, ME. University of Maine.
**37**	Brignon, J.-M., M. Lejart, M. Nexer, S. Michel, A. Quentric, and L. Thiebaud. 2022. A risk-based method to prioritize cumulative impacts assessment on marine biodiversity and research policy for offshore wind farms in France. Environmental Science & Policy 128:264-276.
**38**	Brown-Saracino, J., C. Smith, and P. Gilman. 2013. Mid-Atlantic Marine Wildlife Surveys, Modeling, and Data: Workshop to Establish Coordination & Communication. Page 55 in Workshop to Establish Coordination & Communication.
**39**	Brust, V., and O. Hüppop. 2022. Underestimated scale of songbird offshore migration across the south-eastern North Sea during autumn. Journal of Ornithology 163:51-60.
**40**	Buckland, T. S., L. M. Burt, A. E. Rexstad, M. Mellor, E. A. Williams, and R. Woodward. 2012. Aerial surveys of seabirds: the advent of digital methods. Journal of Applied Ecology 49:960-967.
**41**	Bullman, R., and S. Shaylon. 2012. Analysis of the Argyll Array Turbine Layout Iterations in Relation to Ornithological Constraints. RPS group.

**42**	Bureau of Ocean Energy, M. 2021. Commercial Wind Lease Issuance and Site Assessment Activities on the Atlantic Outer Continental Shelf Offshore North Carolina Draft Supplemental Environmental Assessment. Bureau of Ocean Energy Management (BOEM), U.S. Department of the Interior.
**43**	Bureau of Ocean Energy, M. 2022. Sunrise Wind Draft Environmental Impact Statement (DEIS). Bureau of Ocean Energy Management (BOEM).
**44**	Burger, J., C. Gordon, J. Lawrence, J. Newman, G. Forcey, and L. Vlietstra. 2011. Risk evaluation for federally listed (roseate tern, piping plover) or candidate (red knot) bird species in offshore waters: A first step for managing the potential impacts of wind facility development on the Atlantic Outer Continental Shelf. Renewable Energy 36:338-351.
**45**	Burger, J., J. L. Niles, R. R. Porter, D. A. Dey, S. Koch, and C. Gordon. 2012. Using a shore bird (red knot) fitted with geolocators to evaluate a conceptual risk model focusing on offshore wind. Renewable Energy 43:370-377.
**46**	Burt, L., E. Rexstad, and S. Buckland. 2009. Comparison of Visual and Digital Aerial Survey Results of Avian Abundance for Round 3, Norfolk Region. University of St Andrews.
**47**	Burton, N., S. Holloway, R. Thewlis, N. Carter, and M. Rehfisch. 2006. The Potential Effects on Birds arising from the Onshore Infrastructure of the Proposed Greater Gabbard Offshore Wind Farm. British Trust for Ornithology (BTO).
**48**	Busch, M., and S. Garthe. 2016. Approaching population thresholds in presence of uncertainty: Assessing displacement of seabirds from offshore wind farms. Environmental Impact Assessment Review 56:31-42.
**49**	Busch, M., A. Kannen, S. Garthe, and M. Jessopp. 2013. Consequences of a cumulative perspective on marine environmental impacts: Offshore wind farming and seabirds at North Sea scale in context of the EU Marine Strategy Framework Directive. Ocean & Coastal Management 71:213-224.
**50**	Butler, A., M. Carroll, K. Searle, M. Bolton, J. Waggitt, P. Evans, M. Rehfisch, B. Goddard, M. Brewer, S. Burthe, and F. Daunt. 2020. Attributing seabirds at sea to appropriate breeding colonies and populations. Marine Scotland Science.
**51**	Camphuysen, C. J. K., J. Shamoun-Baranes, W. Bouten, and S. Garthe. 2012. Identifying ecologically important marine areas for seabirds using behavioural information in combination with distribution patterns. Biological Conservation 156:22-29.
**52**	Canning, S., G. Lye, and D. Kerr. 2013. Analysis of Marine Ecology Monitoring Plan Data from the Robin Rigg Offshore Wind Farm, Scotland (Operational Year 3): Appendix 2-Birds. Natural Power.
**53**	Cantlay, C. J., J. S. Portugal, and R. G. Martin. 2024. Visual fields, foraging and collision vulnerability in gulls (Laridae). Ibis.
**54**	Christel, I., G. Certain, A. Cama, R. D. Vieites, and X. Ferrer. 2013. Seabird aggregative patterns: A new tool for offshore wind energy risk assessment. Marine Pollution Bulletin 66:84-91.
**55**	Christensen, T., J. Hounisen, I. Clausager, and I. Petersen. 2004. Visual and Radar Observations of Birds in Relation to Collision Risk at the Horns Rev Offshore Wind Farm. National Environmental Research Institute (NERI).

*56	Christensen-Dalsgaard, S., J. Bustnes, A. Follestad, G. Systad, J. Eriksen, S. Lorentsen, and T. Anker-Nilssen. 2008. Intersectoral Assessment of Impacts on Seabirds: Baseline Report for a Comprehensive Environmental Management Plan for the Norwegian Sea. Norwegian Institute for Nature Research (NINA).
*57	Christensen-Dalsgaard, S., S. Lorentsen, E. Dahl, A. Follestad, F. Hanssen, and G. Systad. 2010. Marine Wind Farms: Seabirds, White-Tailed Eagles, Eurasian Eagle-Owl and Waders - A Screening of Potential Conflict Areas. Norwegian Institute for Nature Research (NINA).
*58	Christensen-Dalsgaard, S., S. Lorentsen, F. Hanssen, and G. Systad. 2011. Marine Wind Farms and Seabirds: Updated National Screening of Potential Conflict Areas. Norwegian Institute for Nature Research (NINA).
59	Cleasby, R. I., D. E. Wakefield, S. Bearhop, W. T. Bodey, C. S. Votier, and C. K. Hamer. 2015. Three-dimensional tracking of a wide-ranging marine predator: flight heights and vulnerability to offshore wind farms. Journal of Applied Ecology 52:1474-1482.
&60	Consultancy, E. E. 2008. Scroby Sands Ornithological Monitoring: Assessing the impacts of the Scroby Sands Offshore Wind Farm upon Little Tern Sternula albifrons: summary of monitoring programme 2002-2006. ECON Ecological Consultancy.
61	Consulting, N. 2012. Argyll Array Offshore Windfarm Report to Inform a Shadow Appropriate Assessment - Great Northern Diver Potential SPAs. ScottishPower Renewables.
62	Cook, A., E. Humphreys, E. Masden, and N. Burton. 2014. The Avoidance Rates of Collision Between Birds and Offshore Turbines. British Trust for Ornithology (BTO).
63	Cook, A., A. Johnson, L. Wright, and N. Burton. 2012. A Review of Flight Heights and Avoidance Rates of Birds in Relation to Offshore Wind Farms. British Trust for Ornithology (BTO).
64	Cook, A., C. Pollock, M. Bogdanova, G. Clewley, F. Daunt, R. Green, B. Swann, and E. Weston. 2023. Seabird tagging feasibility for the Sectoral Marine Plan for Offshore Wind Energy. Scottish Government.
65	Cook, A., V. Ross-Smith, S. Roos, N. Burton, N. Beale, C. Coleman, H. Daniel, S. Fitzpatrick, E. Rankin, K. Norman, and G. Martin. 2011. Identifying a Range of Options to Prevent or Reduce Avian Collision with Offshore Wind Farms using a UK-Based Case Study. British Trust for Ornithology (BTO).
66	Cook, A., C. Thaxter, J. Davies, R. Green, S. Wischnewski, and P. Boersch-Supan. 2023. Understanding seabird behaviour at sea part 2: improved estimates of collision risk model parameters. Scottish Government.
67	Cook, A., R. Ward, W. Hansen, and L. Larsen. 2018. Estimating Seabird Flight Height using LiDAR. British Trust for Ornithology (BTO).
68	Cook, S. C. P. A., M. E. Humphreys, F. Bennet, A. E. Masden, and H. K. N. Burton. 2018. Quantifying avian avoidance of offshore wind turbines: Current evidence and key knowledge gaps. Marine Environmental Research 140:278-288.
69	Copping, A., L. Hanna, V. B. Cleve, K. Blake, and M. R. Anderson. 2015. Environmental Risk Evaluation System—an Approach to Ranking Risk of Ocean Energy Development on Coastal and Estuarine Environments. Estuaries and Coasts 38:287-302.

**70**	Cranmer, A., R. J. Smetzer, L. Welch, and E. Baker. 2017. A Markov model for planning and permitting offshore wind energy: A case study of radio-tracked terns in the Gulf of Maine, USA. Journal of Environmental Management 193:400-409.
**71**	Danish Energy, A. 2006. Offshore Wind Farms and the Environment: Danish Experiences from Horns Rev and Nysted. Danish Energy Agency.
**72**	Danish Energy, A., D. N. Agency, D. Energy, and Vattenfall. 2013. Danish Offshore Wind Key Environmental Issues - A Follow-up. Danish Energy Agency.
**73**	Daunt, F., Z. Fang, R. Howells, M. Harris, S. Wanless, K. Searle, and D. Elston. 2020. Improving estimates of seabird body mass survival relationships. Marine Scotland Science.
**74**	Davies, G. J., H. P. Boersch-Supan, D. G. Clewley, M. E. Humphreys, J. N. O'Hanlon, J. Shamoun-Baranes, B. C. Thaxter, E. Weston, and P. C. S. A. Cook. 2024. Influence of wind on kittiwake Rissa tridactyla flight and offshore wind turbine collision risk. Marine Biology 171.
**75**	Davies, I., and B. Band. 2012. Turbine Height as a Management Tool for Collision Risk to Birds at Offshore Wind Farms. Page 10 in International Council for the Exploration of the Sea 2012.
***76**	De Sutter, R., and A. Volckaet. 2007. MER Offshore Windmolenpark Bligh Bank Belwind NV. Ecolas NV.
**77**	De Sutter, R., and A. Volckaet. 2007. Belwind Environmental Impact Study: Non-Technical Summary. Ecolas NV.
**78**	Defingou, M., F. Bils, B. Horchler, T. Liesenjohann, and G. Nehls. 2019. PHAROS4MPAs: A Review of Solutions to Avoid and Mitigate Environmental Impacts of Offshore Windfarms. BioConsult SH.
**79**	Degraer, S., and R. Brabant. 2009. Offshore Wind Farms in the Belgian Part of the North Sea: State of the Art After Two Years of Environmental Monitoring. Ghent University.
**80**	Degraer, S., R. Brabant, and B. Rumes. 2010. Offshore Wind Farms in the Belgian Part of the North Sea: Early Environmental Impact Assessment and Spatio-Temporal Variability. Ghent University.
**81**	Degraer, S., R. Brabant, and B. Rumes. 2011. Offshore Wind Farms in the Belgian Part of the North Sea: Selected findings from the baseline and targeted monitoring. Management Unit of the North Sea Mathematical Models (MUMM).
**82**	Degraer, S., R. Brabant, and B. Rumes. 2012. Offshore Wind Farms in the Belgian Part of the North Sea: Heading for an Understanding of Environmental Impacts. Ghent University.
**83**	Degraer, S., R. Brabant, B. Rumes, and L. Vigin. 2016. Environmental impacts of offshore wind farms in the Belgian part of the North Sea: Environmental impact monitoring reloaded. Royal Belgian Institute of Natural Sciences (RBINS).
**84**	Degraer, S., R. Brabant, B. Rumes, and L. Vigin. 2017. Environmental Impacts of Offshore Wind Farms in the Belgian Part of the North Sea: a Continued move towards Integration and Quantification. Royal Belgian Institute of Natural Sciences (RBINS).
**85**	Degraer, S., R. Brabant, B. Rumes, and L. Vigin. 2018. Environmental Impacts of Offshore Wind Farms in the Belgian Part of the North Sea: Assessing and Managing Effect Spheres of Influence. Royal Belgian Institute of Natural Sciences (RBINS).

**86**	Degraer, S., R. Brabant, B. Rumes, and L. Vigin. 2019. Environmental Impacts of Offshore Wind Farms in the Belgian Part of the North Sea: Marking a Decade of Monitoring, Research and Innovation. Royal Belgian Institute of Natural Sciences (RBINS).
**87**	Degraer, S., R. Brabant, B. Rumes, and L. Vigin. 2020. Environmental Impacts of Offshore Wind Farms in the Belgian Part of the North Sea: Empirical Evidence Inspiring Priority Monitoring, Research and Management. Royal Belgian Institute of Natural Sciences (RBINS), Brussels, Belgium.
**88**	Degraer, S., R. Brabant, B. Rumes, and L. Vigin. 2021. Environmental Impacts of Offshore Wind Farms in the Belgian Part of the North Sea: Attraction, avoidance and habitat use at various spatial scales. Royal Belgian Institute of Natural Sciences (RBINS), Brussels, Belgium.
**89**	Degraer, S., R. Brabant, B. Rumes, and L. Vigin. 2022. Environmental Impacts of Offshore Wind Farms in the Belgian Part of the North Sea: Getting ready for offshore wind farm expansion in the North Sea. Royal Belgian Institute of Natural Sciences (RBINS), Brussels, Belgium.
**90**	Degraer, S., R. Brabant, B. Rumes, and L. Vigin. 2024. Environmental Impacts of Offshore Wind Farms in the Belgian Part of the North Sea: Progressive Insights in Changing Species Distribution Patterns Informing Marine Management. Royal Belgian Institute of Natural Sciences (RBINS), Brussels, Belgium.
**91**	Desholm, M. 2005. TADS Investigations of Avian Collision Risk at Nysted Offshore Wind Farm. National Environmental Research Institute (NERI).
**92**	Desholm, M. 2005. Preliminary Investigations of Bird-Turbine Collisions at Nysted Offshore Wind Farm and Final Quality Control of Thermal Animal Detection System (TADS). National Environmental Research Institute (NERI).
**93**	Desholm, M. 2006. Wind Farm Related Mortality Among Avian Migrants - A Remote Sensing Study and Model Analysis. PhD Thesis. National Environmental Research Institute (NERI).
**94**	Desholm, M. 2009. Avian sensitivity to mortality: Prioritising migratory bird species for assessment at proposed wind farms. Journal of Environmental Management 90:2672-2679.
**95**	Desholm, M., and J. Kahlert. 2005. Avian collision risk at an offshore wind farm. Biology Letters 1:296-298.
**96**	Desholm, M., J. Kahlert, I. Petersen, and I. Clausager. 2003. Base-Line Investigations of Birds in Relation to an Offshore Wind Farm at Rødsand: Results and Conclusions, 2002. ENERGI E2.
**97**	Dewar, R. 2011. Argyll Array Offshore Wind Farm: Habitat Regulations Assessment - Screening for Likely Significant Effects. RPS group.
**98**	Díaz, H., and G. C. Soares. 2022. A novel multi-criteria decision-making model to evaluate floating wind farm locations. Renewable Energy 185:431-454.
**99**	Díaz, H., A. P. Teixeira, and G. C. Soares. 2022. Application of Monte Carlo and Fuzzy Analytic Hierarchy Processes for ranking floating wind farm locations. Ocean Engineering 245:110453.
**100**	Dierschke, V., M. Frederiksen, G. Glemarec, N. Markones, N. Oliveira, M. Parsons, and N. Vanermen. 2023. Joint OSPAR/HELCOM/ICES Working Group on Seabirds (JWGBIRDS; Outputs from 2022 Meeting). International Council for the Exploration of the Sea (ICES).

**101**	Dierschke, V., W. R. Furness, and S. Garthe. 2016. Seabirds and offshore wind farms in European waters: Avoidance and attraction. Biological Conservation 202:59-68.
***102**	Dirksen, S., A. Spaans, J. Van der Winden, and L. Van den Bergh. 1997. Nocturnal flight patterns and altitudes of diving ducks in the IJsselmeer area. Limosa 71:57-68.
**103**	Dong, E. 2013. Burbo Bank Extension Offshore Wind Farm Volume 2: ES Biological Environment. ABP Marine Environmental Research Ltd (ABPmer).
**104**	Duckworth, J., J. Green, F. Daunt, L. Johnson, P. Lehikoinen, D. Okill, A. Petersen, I. Petersen, R. Väisänen, J. Williams, S. William, and S. O'Brien. 2020. Red-throated Diver Energetics Project: Preliminary Results from 2018/19. Joint Nature Conservation Committee (JNCC).
**105**	E.On. 2012. Rampion Offshore Wind: Environmental Statement. E.ON.
**106**	Ellis, I., and R. Ward. 2013. Burbo Bank Extension Offshore Wind Farm: Red-throated Diver Displacement. NIRAS.
**107**	Engineers, U. S. A. C. o. 2013. Deepwater Wind Block Island Environmental Assessment and Statement of Findings. US Army Corps of Engineers.
**108**	Environmental Resource, M. 2012. East Anglia ONE Offshore Windfarm: Environmental Statement. ScottishPower Renewables.
**109**	Erp, V. A. J., E. E. Loon, J. K. Camphuysen, and J. Shamoun-Baranes. 2021. Temporal Patterns in Offshore Bird Abundance During the Breeding Season at the Dutch North Sea Coast.
**110**	Ess Group, I. 2021. Maryland Offshore Wind Construction and Operations Plan: Appendix II N1 - Avian Risk Assessment. Bureau of Ocean Energy Management (BOEM).
**111**	Fauchald, P., S. M. V. Ollus, M. Ballesteros, A. Breistøl, S. Christensen-Dalsgaard, S. Molværsmyr, A. Tarroux, H. G. Systad, and B. Moe. 2024. Mapping seabird vulnerability to offshore wind farms in Norwegian waters. Frontiers in Marine Science 11.
**112**	Fijn, C. R., L. L. Govers, D. Lutterop, P. R. Middelveld, and V. S. A. R. Bemmelen. 2022. Evidence of Nocturnal Migration over Sea and Sex-Specific Migration Distance of Dutch Black-Headed Gulls. Ardea 110.
**113**	Fijn, C. R., L. K. Krijgsveld, M. J. M. Poot, and S. Dirksen. 2015. Bird movements at rotor heights measured continuously with vertical radar at a <scp>D</scp>utch offshore wind farm. Ibis 157:558-566.
**114**	Flanders, N., B. Gardner, K. Winiarski, P. Paton, T. Allison, and A. O'Connell. 2015. Key seabird areas in southern New England identified using a community occupancy model. Marine Ecology Progress Series 533:277-290.
**115**	Furness, B., and H. Wade. 2012. Vulnerability of Scottish Seabirds to Offshore Wind Turbines. MacArthur Green.
**116**	Furness, W. R., S. Garthe, M. Trinder, J. Matthiopoulos, S. Wanless, and J. Jeglinski. 2018. Nocturnal flight activity of northern gannets Morus bassanus and implications for modelling collision risk at offshore wind farms. Environmental Impact Assessment Review 73:1-6.
**117**	Furness, W. R., M. H. Wade, and A. E. Masden. 2013. Assessing vulnerability of marine bird populations to offshore wind farms. Journal of Environmental Management 119:56-66.

**118**	Galparsoro, I., I. Menchaca, M. J. Garmendia, Á. Borja, D. A. Maldonado, G. Iglesias, and J. Bald. 2022. Reviewing the ecological impacts of offshore wind farms. npj Ocean Sustainability 1.
**119**	Garthe, S., and O. Hüppop. 2004. Scaling possible adverse effects of marine wind farms on seabirds: developing and applying a vulnerability index. Journal of Applied Ecology 41:724-734.
**120**	Garthe, S., N. Markones, and A.-M. Corman. 2017. Possible impacts of offshore wind farms on seabirds: a pilot study in Northern Gannets in the southern North Sea. Journal of Ornithology 158:345-349.
**121**	Garthe, S., V. Peschko, U. Kubetzki, and A.-M. Corman. 2017. Seabirds as samplers of the marine environment – a case study of northern gannets. Ocean Science 13:337-347.
**122**	Garthe, S., N. Sonntag, P. Schwemmer, and V. Dierschke. 2007. Estimation of seabird numbers in the German North Sea throughout the annual cycle and their biogeographic importance. VOGELWELT-BERLIN- 128:163.
**123**	Geo-Marine, I. 2010. Ocean Wind Power Ecological Baseline Studies Final Report - Volume 2: Avian Studies. Geo-Marine Inc.
**124**	Gill, J., D. Sales, S. Pinder, and R. Salazar. 2008. Kentish Flats Wind Farm Fifth Ornithological Monitoring Report. Environmentally Sustainable Systems, Ltd.
**125**	Gill, J. P., A. Mitchell, and S. Pinder. 2007. Annual FEPA Monitoring Report (2005-6) Rhyl Flats Offshore Wind Farm Interim Ornithology Chapter. Environmentally Sustainable Systems, Ltd.
**126**	Gkeka-Serpetsidaki, P., and T. Tsoutsos. 2022. A methodological framework for optimal siting of offshore wind farms: A case study on the island of Crete. Energy 239:19.
**127**	Goodale, W. M., and A. Milman. 2020. Assessing Cumulative Exposure of Northern Gannets to Offshore Wind Farms. Wildlife Society Bulletin 44:252-259.
**128**	Goodale, W. M., A. Milman, and R. C. Griffin. 2019. Assessing the cumulative adverse effects of offshore wind energy development on seabird foraging guilds along the East Coast of the United States. Environmental Research Letters 14:074018.
**129**	Gordon, C. 2011. New Insights and New Tools Regarding Risk to Roseate Terns, Piping Plovers, and Red Knots from Wind Facility Operations on the Atlantic Outer Continental Shelf. Normandeau Associates Inc.
**~130**	Gordon, C., and W. Erickson. 2016. Appendix L-1 WEST Summary of Risks to Birds and Bats. Western Ecosystems Technology Inc (WEST).
**131**	Gordon, C., and W. Erickson. 2016. Project Icebreaker Bird and Bat Risk Analysis. Western Ecosystems Technology Inc (WEST), Cleveland, Ohio.
**~132**	Gordon, C., A. Matteson, J. Stucker, and R. Good. 2018. Appendix L-2 WEST Bird and Bat Annual Report 2018. Western Ecosystems Technology Inc (WEST).
**133**	Gordon, C., and C. Nations. 2016. Collision Risk Model for "rufa" Red Knots (Calidris canutus rufa) Interacting with a Proposed Offshore Wind Energy Facility in Nantucket Sound, Massachusetts. Western Ecosystems Technology Inc (WEST).

**134**	Goyert, F. H., B. Gardner, R. Sollmann, R. R. Veit, T. A. Gilbert, E. E. Connelly, and A. K. Williams. 2016. Predicting the offshore distribution and abundance of marine birds with a hierarchical community distance sampling model. Ecological Applications 26:1797-1815.
**135**	Green, M. W. R., H. K. N. Burton, and S. C. P. A. Cook. 2019. Migratory movements of British and Irish Common Shelduck Tadorna tadorna: a review of ringing data and a pilot tracking study to inform potential interactions with offshore wind farms in the North Sea. Ringing & Migration 34:71-83.
**136**	Griffin, L., E. Rees, and B. Hughes. 2010. Whooper Swan Cygnus cygnus Migration in Relation to Offshore Wind Farms. Page 3 in British Ornithologists' Union Annual Conference (BOU 2010).
**137**	group, R. P. S. 2013. Hornsea Offshore Wind Farm Project One: Environmental Statement: Volume 5 - Offshore Annexes Chapter 5.5.1: Ornithology Technical Report. SMart Wind.
**138**	Grover, W. 2023. Offshore Wind Energy and Seabird Collision Vulnerability in California. Master's Thesis. University of San Francisco.
***139**	Guillemette, M., J. Larsen, and I. Clausager. 1997. Effect of Tunø Knob Windpark on Birds. Danish Ministry of the Environment.
**140**	Guillemette, M., J. Larsen, and I. Clausager. 1998. Impact Assessment of an Off-shore Wind Park on Sea Ducks. National Environmental Research Institute (NERI).
**141**	Guillemette, M., J. Larsen, and I. Clausager. 1999. Assessing the Impact of the Tunø Knob Wind Park on Sea Ducks: The Influence of Food Resources. Danish Ministry of the Environment.
**142**	Guillemette, M., and J. K. Larsen. 2002. POSTDEVELOPMENT EXPERIMENTS TO DETECT ANTHROPOGENIC DISTURBANCES: THE CASE OF SEA DUCKS AND WIND PARKS. Ecological Applications 12:868-877.
**143**	Gușatu, F. L., S. Menegon, D. Depellegrin, C. Zuidema, A. Faaij, and C. Yamu. 2021. Spatial and temporal analysis of cumulative environmental effects of offshore wind farms in the North Sea basin. Scientific Reports 11.
**144**	Halpin, P. 2016. Data to Decision in a Dynamic Ocean: Robust Species Distribution Models and Spatial Decision Frameworks. PhD Thesis. Duke University.
**145**	Harvey, H. T., and Associates. 2020. Existing Conditions and Potential Environmental Effects. HT Harvey and Associates.
**146**	Harwood, P. J. A., R. M. Perrow, and J. R. Berridge. 2018. Use of an optical rangefinder to assess the reliability of seabird flight heights from boat-based surveyors: implications for collision risk at offshore wind farms. Journal of Field Ornithology 89:372-383.
**147**	Harwood, P. J. A., R. M. Perrow, J. R. Berridge, L. M. Tomlinson, and R. E. Skeate. 2017. Unforeseen Responses of a Breeding Seabird to the Construction of an Offshore Wind Farm. Pages 19-41 Wind Energy and Wildlife Interactions.
**148**	Hatch, J., and S. Brault. 2007. Collision Mortalities at Horseshoe Shoal of Bird Species of Special Concern. Cape Wind Associates.

**149**	Heinänen, S., R. Žydelis, M. Dorsch, G. Nehls, and H. Skov. 2017. High-resolution sea duck distribution modeling: Relating aerial and ship survey data to food resources, anthropogenic pressures, and topographic variables. The Condor 119:175-190.
**150**	Heinänen, S., R. Žydelis, B. Kleinschmidt, M. Dorsch, C. Burger, J. Morkūnas, P. Quillfeldt, and G. Nehls. 2020. Satellite telemetry and digital aerial surveys show strong displacement of red-throated divers (Gavia stellata) from offshore wind farms. Marine Environmental Research 160:104989.
**151**	Hexter, R. 2009. High Resolution Video Survey of Seabirds and Mammals in the Rhyl Flats Area. HiDef Aerial Surveying Ltd.
**152**	HiDef Aerial Surveying, L. 2016. Kincardine Offshore Wind Farm Environmental Statement Appendix B: Aerial Surveys Report. Kincardine Offshore Wind Ltd.
**153**	Hill, R., K. Hill, R. Aumüller, A. Schulz, T. Dittmann, C. Kulemeyer, and T. Coppack. 2014. Of birds, blades and barriers: Detecting and analysing mass migration events at alpha ventus. Pages 111-131 in M. Federal, A. Hydrographic, N. C. Federal Ministry for the Environment, and S. Nuclear, editors. Ecological Research at the Offshore Windfarm alpha ventus: Challenges, Results and Perspectives. Springer Fachmedien Wiesbaden, Wiesbaden.
**154**	Horswill, C., H. S. O'Brien, and A. R. Robinson. 2017. Density dependence and marine bird populations: are wind farm assessments precautionary? Journal of Applied Ecology 54:1406-1414.
**155**	Horton, G. 2014. Hornsea Offshore Wind Farm Final Environmental Assessment. UK Department of Energy and Climate Change (DECC).
**156**	Humphreys, E., S. Cook, and N. Burton. 2015. Collision, Displacement and Barrier Effect Concept Note. British Trust for Ornithology (BTO).
**157**	Hüppop, O., J. Dierschke, K. M. Exo, E. Fredrich, and R. Hill. 2006. Bird migration studies and potential collision risk with offshore wind turbines. Ibis 148:90-109.
**158**	Hüppop, O., and G. Hilgerloh. 2012. Flight call rates of migrating thrushes: effects of wind conditions, humidity and time of day at an illuminated offshore platform. Journal of Avian Biology 43:85-90.
**159**	Hüppop, O., K. Hüppop, J. Dierschke, and R. Hill. 2016. Bird collisions at an offshore platform in the North Sea. Bird Study 63:73-82.
**160**	Inc, E. S. S. G. 2006. Tern Observations Near Monomoy Island. ESS Group Inc.
**161**	Inc, E. S. S. G. 2006. Long-tailed Duck Report, Winter 2005-2006. ESS Group Inc.
**162**	Inc, E. S. S. G. 2006. Potential Impacts to Predator-Prey Relationships as a Result of the Proposed Cape Wind Project in Nantucket Sound. Cape Wind Associates.
**163**	International, M. a. D. C. 2013. Windpark SeaStar Milieueffectenrapport.
***#164**	International, M. a. D. C. 2014. Windpark Mermaid Milieueffectenrapport.
**165**	Jarvis, C. 2005. An Evaluation of the Wildlife Impacts of Offshore Wind Development Relative to Fossil Fuel Power Production. Master's Thesis. University of Delaware.

**166**	Jenner, C. 2014. Hornsea Offshore Wind Farm Project One: UPDATED IN-COMBINATION AUK DISPLACEMENT NOTE. SMart Wind, London, UK.
**&167**	Jervis, L., S. Warford, A. Goulding, N. Dominguez Alvarez, M. Arundale, A. Thompson-Allsop, R. Worrall, J. Robinson Willmott, S. Clough, and S. Sweeney. 2019. Ornithological and Marine Fauna Aerial Survey Results of Lease Area OCS-A 0512, Annual Report: November 2017 to October 2018. Normandeau Associates Inc.
**168**	Jervis, L., S. Warford, A. Goulding, J. Robinson Willmott, S. Clough, and S. Sweeney. 2019. Ornithological and Marine Fauna Aerial Survey Results, Annex 1: Summer 2016 to Spring 2017. Normandeau Associates Inc.
**&169**	Jervis, L., S. Warford, J. Willmott, S. Clough, and R. Buisson. 2018. Ornithological and Marine Fauna Aerial Survey Results of Lease Area OCS-A 0512 Equinor Quarterly Report: February 2018 to April 2018. Normandeau Associates Inc.
**170**	Johnson, C., C. Currier, J. Howar, M. Elliott, C. Rockwood, and J. Jahncke. 2022. Environmental Data Catalog for the Morro Bay Wind Energy Area. SeaJay Environmental.
**171**	Johnson, C., C. Currier, J. Howar, M. Elliott, C. Rockwood, and J. Jahncke. 2022. Environmental Data Catalog for the Humboldt Wind Energy Area. Point Blue Conservation Science.
**172**	Johnston, A., P. C. S. A. Cook, J. L. Wright, M. E. Humphreys, and K. H. N. Burton. 2014. Modelling flight heights of marine birds to more accurately assess collision risk with offshore wind turbines. Journal of Applied Ecology 51:31-41.
**173**	Johnston, D., C. Thaxter, P. Boersch-Supan, E. Humphreys, W. Bouten, G. Clewley, E. Scragg, E. Masden, L. Barber, G. Conway, N. Clark, N. Burton, and A. Cook. 2022. Investigating avoidance and attraction responses in lesser black-backed gulls Larus fuscus to offshore wind farms. Marine Ecology Progress Series 686:187-200.
**174**	Jones, R., C. Peggie, D. Fouracre, P. Cropper, J. Finney, and S. Wilson. 2015. Humber Gateway: Bird Detection Radar Pink-footed Goose Monitoring Autumn 2015. Institute of Estuarine and Coastal Studies (IECS).
**175**	Jongbloed, R., J. Tamis, J. van der Wal, P. de Vries, A. Grundlehner, and G. Piet. 2023. Quick scan of cumulative impacts on the North Sea biodiversity: with a focus on selected species in relation to future developments in offshore wind energy. Wageningen University and Research Centre.
**176**	Kahlert, J., I. Petersen, A. Fox, M. Desholm, and I. Clausager. 2004. Investigations of Birds During Construction and Operation of Nysted Offshore Wind Farm at Rødsand. National Environmental Research Institute (NERI).
**177**	Kaiser, J. M., M. Galanidi, A. D. Showler, J. A. Elliott, G. W. R. Caldow, S. I. E. Rees, A. R. Stillman, and J. W. Sutherland. 2006. Distribution and behaviour of Common Scoter <i>Melanitta nigra</i> relative to prey resources and environmental parameters. Ibis 148:110-128.
**178**	Kaiser, M., A. Elliott, M. Galanidi, E. Ivor, S. Rees, R. Caldow, R. Stillman, W. Sutherland, and D. Showler. 2002. Predicting the Displacement of Common Scoter Melanitta nigra from Benthic Feeding Areas Due to Offshore Windfarms. Bangor University.

179	Kelsey, C. E., J. J. Felis, M. Czapanskiy, M. D. Pereksta, and J. Adams. 2018. Collision and displacement vulnerability to offshore wind energy infrastructure among marine birds of the Pacific Outer Continental Shelf. Journal of Environmental Management 227:229-247.
180	Kennedy, L. 2013. Visual Observations for Birds, Turtles, and Marine Mammals at the University of Maine Test Site near Monhegan, Maine. University of Maine, Monhegan, Maine.
181	Kinlan, B., A. Winship, T. White, and J. Christensen. 2016. Modeling At-Sea Occurrence and Abundance of Marine Birds to Support Atlantic Marine Renewable Energy Planning: Phase 1 Report. US Department of Commerce (DOC).
182	Köller, J., J. Köppel, and W. Peters. 2006. Offshore Wind Energy: Research on Environmental Impacts. Springer Berlin Heidelberg.
&183	Krijgsveld, K., R. Fijn, C. Heunks, P. van Horssen, J. de Fouw, M. Collier, M. Poot, E. Meesters, and S. Dirksen. 2008. Effect Studies Offshore Wind Farm Egmond aan Zee: Progress Report on Fluxes and Behaviour of Flying Birds. Bureau Waardenburg bv.
184	Krijgsveld, K., R. Fijn, and R. Lensink. 2015. Occurrence of peaks in songbird migration at rotor heights of offshore wind farms in the southern North Sea. Bureau Waardenburg bv.
&185	Krijgsveld, K., R. Fljn, M. Japink, P. van Horssen, C. Heunks, M. Collier, M. Poot, D. Beuker, and S. Dirksen. 2011. Effect Studies Offshore Wind Farm Egmond aan Zee - Final Report on Fluxes, Flight Altitudes and Behaviour of Flying Birds. Bureau Waardenburg bv.
186	Krijgsveld, K., R. Lensink, H. Schekkerman, P. Wiersma, M. Poot, E. Meesters, and S. Dirksen. 2005. Baseline Studies North Sea Wind Farms: Fluxes, Flight Paths and Altitudes of Flying Birds 2003-2004. Alterra - Wageningen UR.
187	Lamb, J., J. Gulka, E. Adams, A. Cook, and A. K. Williams. 2024. A synthetic analysis of post-construction displacement and attraction of marine birds at offshore wind energy installations. Environmental Impact Assessment Review 108:107611.
188	Lane, V. J., R. Jeavons, Z. Deakin, B. R. Sherley, J. C. Pollock, J. R. Wanless, and C. K. Hamer. 2020. Vulnerability of northern gannets to offshore wind farms; seasonal and sex-specific collision risk and demographic consequences. Marine Environmental Research 162:105196.
189	Langston, R. 2010. Offshore Wind Farms and Birds: Round 3 Zones, Extensions to Round 1 & Round 2 Sites & Scottish Territorial Waters. Royal Society for the Protection of Birds (RSPB).
190	Langston, R., E. Teuten, and A. Butler. 2013. Foraging Ranges of Northern Gannets in Relation to Proposed Offshore Wind Farms in the UK: 2010-2012. Royal Society for the Protection of Birds (RSPB).
191	Layton-Matthews, K., L. Buckingham, E. Critchley, A. Nilsson, V. Ollus, M. Ballesteros, S. Christensen-Dalsgaard, N. Dehnhard, P. Fauchald, F. Hanssen, M. Helberg, E. Masden, R. May, H. Sandvik, A. Tarroux, and T. Reiertsen. 2023. Development of a Cumulative Impact Assessment tool for birds in Norwegian Offshore Waters: Trollvind OWF as a case study. Norwegian Institute for Nature Research (NINA), Tromsø, Norway.
192	Leirness, J. 2021. Modeling At-Sea Density of Marine Birds to Support Renewable Energy Planning on the Pacific Outer Continental Shelf of the Contiguous United States. CSS, Inc.

**193**	Lemos, A. C., M. Hernández, C. Vilardo, A. R. Phillips, L. Bugoni, and I. Sousa-Pinto. 2023. Environmental assessment of proposed areas for offshore wind farms off southern Brazil based on ecological niche modeling and a species richness index for albatrosses and petrels. Global Ecology and Conservation 41:e02360.
**194**	Leopold, M., M. Boonman, M. Collier, N. Davaasuren, R. Fijn, A. Gyimesi, J. de Jong, R. Jongbloed, B. Poerink, K. Krijgsveld, S. Lagerveld, R. Lensink, M. Poot, J. van der Wal, and M. Scholl. 2015. Framework for Assessing Ecological and Cumulative Effects of Offshore Wind Farms: A First Approach to Deal with Cumulative Effects on Birds and Bats of Offshore Wind Farms and Other Human Activities in the Southern North Sea. IMARES - Wageningen UR.
**&195**	Leopold, M., M. Boonman, M. Collier, N. Davaasuren, R. Fijn, A. Gyimesi, J. Jong, R. Jongbloed, B. Poerink, J. Kleyheeg-Hartman, K. Krijgsveld, S. Lagerveld, R. Lensink, M. Poot, J. van der Wal, and M. Scholl. 2015. A first approach to deal with cumulative effects on birds and bats of offshore wind farms and other human activities in the Southern North Sea. IMARES - Wageningen UR.
**196**	Leopold, M., and K. Camphuysen. 2009. Did the Pile Driving during the Construction of the Offshore Wind Farm Egmond aan Zee, the Netherlands, Impact Local Seabirds? , IMARES - Wageningen UR.
**197**	Leopold, M., K. Camphuysen, C. ter Braak, E. Dijkman, K. Kersting, and S. van Lieshout. 2004. Baseline Studies North Sea Wind Farms: Lot 5 Marine Birds in and Around the Future Sites Nearshore Windfarm (NSW) and Q7. Alterra - Wageningen UR.
**198**	Leopold, M., M. Collier, A. Gyimesi, R. Jongbloed, M. Poot, J. van der Wal, and M. Scholl. 2015. Iteration cycle: Dealing with peaks in counts of birds following active fishing vessels when assessing cumulative effects of offshore wind farms and other human activities in the Southern North Sea. IMARES - Wageningen UR.
**199**	Leopold, M., E. Dijkman, and L. Teal. 2011. Local Birds in and around the Offshore Wind Farm Egmond aan Zee (OWEZ). IMARES - Wageningen UR.
**200**	Leopold, M., R. van Bemmelen, and A. Zuur. 2013. Responses of Local Birds to the Offshore Wind Farms PAWP and OWEZ off the Dutch mainland coast. IMARES - Wageningen UR.
**201**	Lewis, M., G. Lye, C. Pendlebury, and R. Walls. 2012. Population Sizes of Seabirds breeding in Scottish Special Protection Areas. Natural Power.
**202**	Lieske, J. D., M. L. Tranquilla, A. R. Ronconi, and S. Abbott. 2020. “Seas of risk”: Assessing the threats to colonial-nesting seabirds in Eastern Canada. Marine Policy 115:103863.
**203**	Lieske, J. D., M. L. Tranquilla, R. Ronconi, and S. Abbott. 2019. Synthesizing expert opinion to assess the at-sea risks to seabirds in the western North Atlantic. Biological Conservation 233:41-50.
***204**	Lorensten, S., S. Christensen-Dalsgaard, A. Follestad, M. Langset, R. May, E. Dahl, and Ø. Hamre. 2012. Strategic Impact Assessment of Offshore Windpower in Norway - Impacts on Seabirds. Norwegian Institute for Nature Research (NINA).
**205**	Loring, H. P., W. C. P. Paton, E. J. Osenkowski, G. S. Gilliland, L. J. P. Savard, and R. S. Mcwilliams. 2014. Habitat use and selection of black scoters in southern New England and siting of offshore wind energy facilities. The Journal of Wildlife Management 78:645-656.

**206**	Loring, P. 2016. Evaluating Digital VHF Technology to Monitor Shorebird and Seabird Use of Offshore Wind Energy Areas in the Western North Atlantic. PhD Thesis. University of Massachusetts.
**207**	Loring, P., A. Lenske, D. McLaren, M. Aikens, A. Anderson, Y. Aubry, E. Dalton, A. Dey, C. Friis, D. Hamilton, R. Holberton, D. Kriensky, D. Mizrahi, L. Niles, K. Parkins, J. Paquet, F. Sanders, A. Smith, Y. Turcotte, A. Vitz, and P. Smith. 2021. Tracking Movements of Migratory Shorebirds in the U.S. Atlantic Outer Continental Shelf Region. US Fish and Wildlife Service (USFWS).
**208**	Ltd, A. 2010. London Array Offshore Wind Farm Project - Aerial survey methods, data collection and statistical analysis. APEM Ltd.
**&209**	Ltd, A. 2012. London Array Offshore Wind Farm Project - Aerial Bird Survey Report Winter 2010/11 & 2011/12 (Zones 1 & 2). APEM Ltd.
**&210**	Ltd, A. 2013. London Array Offshore Wind Farm: Ornithology Aerial Survey Report 2012-2013. APEM Ltd.
**&211**	Ltd, A. 2016. London Array Offshore Wind Farm: Ornithology Aerial Survey Report 2013 / 2014. APEM Ltd.
**~212**	Ltd, A., and N. A. Inc. 2022. Kitty Hawk North Wind COP Appendix S: Ornithological and Marine Fauna Aerial Survey Results. Bureau of Ocean Energy Management (BOEM).
**213**	Maclean, D. M. I., M. M. Rehfisch, H. Skov, and B. C. Thaxter. 2013. Evaluating the statistical power of detecting changes in the abundance of seabirds at sea. Ibis 155:113-126.
**214**	Malcolm, F., G. Lye, and M. Lewis. 2012. Population Trends of Breeding Seabird Colonies in Scottish SPAs. Natural Power.
**215**	Manola, I., M. Bradarić, R. Groenland, R. Fijn, W. Bouten, and J. Shamoun-Baranes. 2020. Associations of Synoptic Weather Conditions With Nocturnal Bird Migration Over the North Sea. Frontiers in Ecology and Evolution 8.
**216**	Mardik, F., M. Leopold, and H. Verdaat. 2018. Pilot field study: observations from a fixed platform on occurrence and behaviour of common guillemots and other seabirds in offshore wind farm Luchterduinen. Wageningen University and Research Centre.
**217**	Marine, S. 2023. 5th ScotMER Symposium: Ornithology 1.in 5th Symposium of the Scottish Marine Energy Research Programme (ScotMER).
**218**	Marine, S. 2023. 5th ScotMER Symposium: Ornithology 2.in 5th Symposium of the Scottish Marine Energy Research Programme (ScotMER).
**&219**	MarineSpace, L. 2015. London Array Offshore Wind Farm Year 1 Post-Construction Monitoring Report. MarineSpace Ltd.
**220**	Masden, A. E., S. C. P. A. Cook, A. Mccluskie, W. Bouten, H. K. N. Burton, and B. C. Thaxter. 2021. When speed matters: The importance of flight speed in an avian collision risk model. Environmental Impact Assessment Review 90:106622.
**221**	Masden, A. E., T. D. Haydon, D. A. Fox, and W. R. Furness. 2010. Barriers to movement: Modelling energetic costs of avoiding marine wind farms amongst breeding seabirds. Marine Pollution Bulletin 60:1085-1091.

**222**	Masden, A. E., R. Reeve, M. Desholm, D. A. Fox, W. R. Furness, and T. D. Haydon. 2012. Assessing the impact of marine wind farms on birds through movement modelling. Journal of The Royal Society Interface 9:2120-2130.
**223**	Mateos-Rodriguez, M., L. Thomas, and G. Arroyo. 2012. The Development and Use of a Method to Fill Time Gaps in Migration Counts. The Condor 114:513-522.
***224**	Matthieu, F., C. Benjamin, L. Franck, and E. Ouvrard. 2014. Diagnostic environnemental 2013-2014 pour le groupe avifaune et évaluation du risque d'impact dans le cadre du projet de parc éolien en mer de Saint- Nazaire.
**225**	May, J. 2005. Post-Construction Results from The North Hoyle Offshore Wind Farm. Page 1 in Copenhagen Offshore Wind International Conference 2005.
**226**	McGovern, S., B. Goddard, and M. Rehfisch. 2016. Assessment of Displacement Impacts of Offshore Windfarms and Other Human Activities on Red-Throated Divers and Alcids. APEM Ltd.
***#227**	McGregor, R. 2014. Parc éolien en mer de St Nazaire : Analyse complémentaire des impacts sur le Goéland marin (Larus marinus). Natural Power.
**228**	McGregor, R., M. Trinder, and N. Goodship. 2022. Assessment of compensatory measures for impacts of offshore windfarms on seabirds. Natural England.
**229**	Meattey, E. D., R. S. Mcwilliams, C. W. P. Paton, C. Lepage, G. S. Gilliland, L. Savoy, H. G. Olsen, and E. J. Osenkowski. 2019. Resource selection and wintering phenology of White-winged Scoters in southern New England: Implications for offshore wind energy development. The Condor 121.
**230**	Mellor, M., and M. Maher. 2008. Full Scale Trial of High Definition Video Survey for Offshore Windfarm Sites. HiDef Aerial Surveying Ltd.
**231**	Mendel, B., J. Kotzerka, J. Sommerfeld, H. Schwemmer, N. Sonntag, and S. Garthe. 2014. Effects of the alpha ventus offshore test site on distribution patterns, behaviour and flight heights of seabirds. Pages 95-110 in M. Federal, A. Hydrographic, N. C. Federal Ministry for the Environment, and S. Nuclear, editors. Ecological Research at the Offshore Windfarm alpha ventus: Challenges, Results and Perspectives. Springer Fachmedien Wiesbaden, Wiesbaden.
**232**	Mendel, B., P. Schwemmer, V. Peschko, S. Müller, H. Schwemmer, M. Mercker, and S. Garthe. 2019. Operational offshore wind farms and associated ship traffic cause profound changes in distribution patterns of Loons (Gavia spp.). Journal of Environmental Management 231:429-438.
**233**	Menza, C., B. Kinlan, D. Dorfman, M. Poti, and C. Caldow. 2012. A Biogeographic Assessment of Seabirds, Deep Sea Corals and Ocean Habitats of the New York Bight: Science to Support Offshore Spatial Planning. National Oceanic and Atmospheric Administration (NOAA).
**234**	Mercker, M., V. Dierschke, K. Camphuysen, A. Kreutle, N. Markones, N. Vanermen, and S. Garthe. 2021. An indicator for assessing the status of marine-bird habitats affected by multiple human activities: A novel statistical approach. Ecological Indicators 130:14.
**235**	Mikami, K., K. Kazama, T. M. Kazama, and Y. Watanuki. 2022. Mapping the collision risk between two gull species and offshore wind turbines: Modelling and validation. Journal of Environmental Management 316:115220.

**236**	Montevecchi, W. A., A. Hedd, M. L. Tranquilla, D. A. Fifield, C. M. Burke, P. M. Regular, G. K. Davoren, S. Garthe, G. J. Robertson, and R. A. Phillips. 2012. Tracking seabirds to identify ecologically important and high risk marine areas in the western North Atlantic. Biological Conservation 156:62-71.
**237**	Morandi, A., S. Berkman, J. Rowe, R. Balouskus, D. Reich, D. Etkin, and C. Moelter. 2018. Environmental Sensitivity and Associated Risk to Habitats and Species Offshore Central California and Hawaii from Offshore Floating Wind Technologies. RPS group, Sacramento, CA, US.
**238**	Murray, L., H. Bloomfield, and L. Skates. 2023. Mapping Environmental Considerations for Marine Spatial Planning in Wales: Methodology. Natural Resources Wales, Bangor, Wales.
**239**	Musial, W., R. Green, S. Courbis, J. Frolich, K. White, R. Austria, and J. Pfeifenberger. 2022. Great Lakes Wind Energy Feasibility Study. National Renewable Energy Laboratory (NREL).
**~240**	Nations, C., and C. Gordon. 2017. Appendix J WEST NEXRAD Analysis: Assessment of Nocturnal Bird Migration Activity from Weather Radar Data for the Proposed Icebreaker Wind Energy Facility, Lake Erie, Ohio. Western Ecosystems Technology Inc (WEST).
**241**	New York Department of, S. 2013. New York Department of State Offshore Atlantic Ocean Study. New York State Department of State.
**242**	Noer, H., T. Christensen, I. Clausager, and I. Petersen. 2000. Effects on Birds of an Offshore Wind Park at Horns Rev: Environmental Impact Assessment. National Environmental Research Institute (NERI).
**243**	Normandeau Associates, I. 2013. High-Resolution Aerial Imaging Surveys of Marine Birds, Mammals, and Turtles on the US Atlantic Outer Continental Shelf - Utility Assessment, Methodology Recommendations, and Implementation Tools. Normandeau Associates Inc.
**&244**	Normandeau Associates, I. 2017. Digital Aerial Baseline Survey of Marine Wildlife in Support of Offshore Wind Energy First Annual Report. Normandeau Associates Inc.
**&245**	Normandeau Associates, I. 2019. Digital Aerial Baseline Survey of Marine Wildlife in Support of Offshore Wind Energy Second Annual Report Fourth Interim Report. Normandeau Associates Inc.
**&246**	Normandeau Associates, I. 2020. Digital Aerial Baseline Survey of Marine Wildlife in Support of Offshore Wind Energy Fifth Interim Report. Normandeau Associates Inc.
**&247**	Normandeau Associates, I. 2020. Digital Aerial Baseline Survey of Marine Wildlife in Support of Offshore Wind Energy Sixth Interim Report. Normandeau Associates Inc.
**&248**	Normandeau Associates, I. 2023. Ornithological and Marine Fauna Aerial Digital Survey: First Annual Report. Normandeau Associates Inc, Gainesville, FL.
**249**	Normandeau Associates, I. 2023. Hudson South 2 E05 Bat and Bird Acoustic Analysis and Results Summary. Normandeau Associates Inc.
**&250**	Normandeau Associates, I. 2023. Ornithological and Marine Fauna Aerial Digital Survey: First Semiannual Report. Normandeau Associates Inc, Gainesville, FL.
**251**	O'Brien, B. 2023. A Geospatial Analysis of Species of Interest in US Atlantic Wind Energy Areas. Master's Thesis. Duke University.

**252**	O'Hanlon, N. J., S. Wischnewski, D. Ewing, K. Newman, C. Gunn, E. L. Jones, M. Newell, A. Butler, M. Quintin, K. Searle, R. Walker, E. M. Humphreys, L. J. Wright, F. Daunt, and R. A. Robinson. 2021. Feasibility study of large-scale deployment of colour-ringing on Black-legged Kittiwake populations to improve the realism of demographic models assessing the population impacts of offshore wind farms. British Trust for Ornithology (BTO), Peterborough, United Kingdom.
**253**	Obane, H., K. Kazama, H. Hashimoto, Y. Nagai, and K. Asano. 2024. Assessing areas suitable for offshore wind energy considering potential risk to breeding seabirds in northern Japan. Marine Policy 160:105982.
**254**	Ocean, W. 2023. Ocean Wind 1 Construction and Operations Plan. PSEG Power, New Jersey, USA.
**~255**	Ørsted, and Eversource. 2021. South Fork Wind Farm Construction and Operations Plan. Ørsted.
**#256**	Parc eolien en mer de St, N. 2020. SAINT-NAZAIRE Bilan environnemental annuel 2020. Parc éolien en mer de Saint-Nazaire.
**257**	Paton, P., C. Cooper-Mullin, S. Kouhi, P. Loring, J. Moore, J. Miller, and G. Potty. 2021. Assessing movements of birds using digitally-coded VHF transmitters: A validation study. University of Rhode Island.
**258**	Pavat, D., A. Harker, G. Humphries, K. Keogan, A. Webb, and K. Macleod. 2023. Consideration of avoidance behaviour of northern gannet (Morus bassanus) in collision risk modelling for offshore wind farm impact assessments (NECR512). HiDef Aerial Surveying Ltd.
**&259**	Pembroke, A., and J. Robinson Willmott. 2021. Digital Aerial Baseline Survey of Marine Wildlife in Support of Offshore Wind Energy: Spatial and Temporal Marine Wildlife Distributions in the New York Offshore Planning Area, Summer 2016–Spring 2019. Normandeau Associates Inc.
**260**	Percival, S. 2010. Kentish Flats Offshore Wind Farm: Diver Surveys 2009-10. Vattenfall.
**261**	Percival, S., T. Percival, and C. Hartley. 2010. Robin Rigg Solway Offshore Wind Farm: Bird Monitoring Programme Report No. 8: construction phase bird surveys October-December 2009 and comparison of construction phase data with previous baseline. Ecology Consulting.
**262**	Perrow, M. 2019. Wildlife and Wind Farms - Conflicts and Solutions, Volume 3. Offshore: Potential Effects. Pelagic Publishing, Exeter, UK.
**263**	Perrow, M., J. Gilroy, E. Skeate, and A. Mackenzie. 2010. Quantifying the Relative Use of Coastal Waters by Breeding Terns: Towards Effective Tools for Planning & Assessing the Ornithological Impact of Offshore Wind Farms. ECON Ecological Consultancy.
**264**	Perrow, M., A. Harwood, E. Praca, E. Skeate, A. Green, and M. Tomlinson. 2013. Little tern Sternula albifrons use of Scroby Sands Offshore Wind Farm in 2013. ECON Ecological Consultancy.
**&265**	Perrow, M., E. Skeate, and M. Tomlinson. 2005. Scroby Sands Ornithological Monitoring: Assessing the potential impact of the proposed wind farm upon Little tern Sterna albifrons: the construction phase 2004. ECON Ecological Consultancy.

**266**	Perrow, R. M., J. J. Gilroy, R. E. Skeate, and L. M. Tomlinson. 2011. Effects of the construction of Scroby Sands offshore wind farm on the prey base of Little tern Sternula albifrons at its most important UK colony. Marine Pollution Bulletin 62:1661-1670.
**267**	Perrow, R. M., R. E. Skeate, and J. J. Gilroy. 2011. Visual tracking from a rigid-hulled inflatable boat to determine foraging movements of breeding terns. Journal of Field Ornithology 82:68-79.
**268**	Perrow, R. M., R. E. Skeate, P. Lines, D. Brown, and L. M. Tomlinson. 2006. Radio telemetry as a tool for impact assessment of wind farms: the case of Little Terns <i>Sterna albifrons</i> at Scroby Sands, Norfolk, UK. Ibis 148:57-75.
**269**	Peschko, V., B. Mendel, M. Mercker, J. Dierschke, and S. Garthe. 2021. Northern gannets (Morus bassanus) are strongly affected by operating offshore wind farms during the breeding season. Journal of Environmental Management 279:111509.
**270**	Peschko, V., B. Mendel, S. Müller, N. Markones, M. Mercker, and S. Garthe. 2020. Effects of offshore windfarms on seabird abundance: Strong effects in spring and in the breeding season. Marine Environmental Research 162:105157.
**271**	Peschko, V., M. Mercker, and S. Garthe. 2020. Telemetry reveals strong effects of offshore wind farms on behaviour and habitat use of common guillemots (Uria aalge) during the breeding season. Marine Biology 167.
**272**	Peschko, V., H. Schwemmer, M. Mercker, N. Markones, K. Borkenhagen, and S. Garthe. 2024. Cumulative effects of offshore wind farms on common guillemots (Uria aalge) in the southern North Sea - climate versus biodiversity? Biodiversity and Conservation 33:949-970.
**273**	Petersen, I. 2005. Bird Numbers and Distributions in the Horns Rev Offshore Wind Farm Area. ELSAM Engineering.
**274**	Petersen, I. 2007. Modelling Total Numbers and Distribution of Common Scoter Melanitta nigra at Horns Rev. Aarhus University.
**275**	Petersen, I., T. Christensen, J. Kahlert, M. Desholm, and A. Fox. 2006. Final Results of Bird Studies at the Offshore Wind Farms at Nysted and Horns Rev, Denmark. Dong Energy.
**276**	Petersen, I., and A. Fox. 2007. Changes in Bird Habitat Utilisation around the Horns Rev 1 Offshore Wind Farm, with Particular Emphasis on Common Scoter. National Environmental Research Institute (NERI).
**277**	Petersen, I., M. Mackenzie, E. Rexstad, M. Wisz, and A. Fox. 2011. Comparing Pre- and Post-Construction Distributions of Long-Tailed Ducks Clangula hyemalis in and around the Nysted Offshore Wind Farm, Denmark: A Quasi-Designed Experiment Accounting for Imperfect Detection, Local Surface Features and Autocorrelation. University of St Andrews.
**278**	Peterson, C. 2011. Risks to Birds and Wildlife from Offshore Wind Farms: BOEMRE NC Task Force. Page 32.
**279**	Pettersson, J. 2005. The Impact of Offshore Wind Farms on Bird Life in Southern Kalmar Sound, Sweden. Lund University.
**280**	Pettersson, J., and J. Fågelvind. 2011. Night Migration of Songbirds and Waterfowl at the Utgrunden Off-Shore Wind Farm: A Radar-Assisted Study in Southern Kalmar Sound. Vindval.

**281**	Pettersson, J., and T. Stalin. 2003. Influence of offshore windmills on migration birds in southeast coast of Sweden. GE Energy AB, Sweden.
**282**	Piet, G., J. Tamis, J. van der Wal, and R. Jongbloed. 2021. Cumulative impacts of wind farms on the North Sea ecosystem. Wageningen University and Research Centre, Wageningen Marine Research.
**283**	Piet, J. G., E. J. Tamis, J. Volwater, D. P. Vries, D. V. T. J. Wal, and H. R. Jongbloed. 2021. A roadmap towards quantitative cumulative impact assessments: Every step of the way. Science of The Total Environment 784:146847.
**284**	Piper, W., G. Kulik, J. Durinck, H. Skov, and S. Leonhard. 2008. Horns Rev II Offshore Wind Farm Monitoring of Migrating Waterbirds. Danish Hydraulic Institute (DHI).
**285**	Plonczkier, P., and C. I. Simms. 2012. Radar monitoring of migrating pink-footed geese: behavioural responses to offshore wind farm development. Journal of Applied Ecology 49:1187-1194.
**286**	Pollock, J. C., V. J. Lane, L. Buckingham, S. Garthe, R. Jeavons, W. R. Furness, and C. K. Hamer. 2021. Risks to different populations and age classes of gannets from impacts of offshore wind farms in the southern North Sea. Marine Environmental Research 171:105457.
**287**	Poot, M., P. van Horssen, M. Collier, R. Lensink, and S. Dirksen. 2011. Effect Studies Offshore Wind Egmond aan Zee: Cumulative Effects on Seabirds - A Modelling Approach to Estimate Effects on Population Levels in Seabirds. Bureau Waardenburg bv.
**288**	Poot, M., P. van Horssen, M. Collier, R. Lensink, and S. Dirksen. 2012. Cumulative Effects of Wind Farms in the Dutch North Sea on Bird Populations. Page 7 in 32nd Annual Meeting of the International Association for Impact Assessment (IAIA 2012).
**289**	Potiek, A., M. Collier, H. Schekkerman, and R. Fijn. 2019. Effects of turbine collision mortality on population dynamics of 13 bird species. Bureau Waardenburg bv.
**290**	Potiek, A., G. J. Ijntema, T. van Kooten, M. Leopold, and M. Collier. 2022. Acceptable Levels of Impact from offshore wind farms on the Dutch Continental Shelf for 21 bird species. Bureau Waardenburg bv.
**291**	Power, N. 2002. Robin Rigg Environmental Statement.
**292**	Programs, B. O. o. R. E. 2022. Biological Assessment: Revolution Wind Farm and Revolution Export Cable - Development and Operation. US Department of the Interior (DOI).
**293**	Programs, B. O. o. R. E. 2023. Revolution Wind Farm and Revolution Wind Export Cable Project Final Environmental Impact Statement (FEIS). BOEM Office of Renewable Energy Programs.
**294**	Programs, B. O. o. R. E. 2023. SouthCoast Wind Draft Environmental Impact Statement. Bureau of Ocean Energy Management (BOEM).
**295**	Rebke, M., V. Dierschke, N. C. Weiner, R. Aumüller, K. Hill, and R. Hill. 2019. Attraction of nocturnally migrating birds to artificial light: The influence of colour, intensity and blinking mode under different cloud cover conditions. Biological Conservation 233:220-227.
**296**	Rehfisch, M., Z. Barrett, L. Brown, R. Buisson, R. Perez-Dominguez, and S. Clough. 2014. Assessing Northern Gannet Avoidance of Offshore Windfarms. APEM Ltd.

**297**	Reid, K., B. G. Baker, and J. E. Woehler. 2023. An ecological risk assessment for the impacts of offshore wind farms on birds in Australia. Austral Ecology 48:418-439.
**298**	Repsol, and G. O. a. G. C. Ltd. 2016. Beatrice Environmental Impact Assessment Report (Consultation Version). Repsol, Aberdeen, Scotland.
***299**	Reynaud, M., E. Le Bouhris, T. Soulard, and Y. Perignon. 2021. FLOATGEN floating wind turbine environmental monitoring report, SEM-REV test site. Ecole Central de Nantes (ECN).
**300**	Ross-Smith, H. V., B. C. Thaxter, A. E. Masden, J. Shamoun-Baranes, K. H. N. Burton, J. L. Wright, M. M. Rehfisch, and A. Johnston. 2016. Modelling flight heights of lesser black-backed gulls and great skuas from GPS: a Bayesian approach. Journal of Applied Ecology 53:1676-1685.
**301**	Rothery, P., I. Newton, and B. Little. 2009. Observations of seabirds at offshore wind turbines near Blyth in northeast England. Bird Study 56:1-14.
**302**	Sadoul, N. 2021. Document cadre de la MR9 Réduire les dérangements de la halte migratoire du Puffin des Baléares. Bretagne Développement Innovation.
**303**	Saunders, S. 2012. Ormonde Offshore Wind Farm 2010 Construction Environmental Monitoring Report. RPS group.
**304**	Schneider, R. S., H. S. Kramer, B. S. Bernstein, B. S. Terrill, G. D. Ainley, and S. Matzner. 2024. Autonomous thermal tracking reveals spatiotemporal patterns of seabird activity relevant to interactions with floating offshore wind facilities. Frontiers in Marine Science 11.
**305**	ScottishPower, R. 2017. East Anglia TWO Offshore Windfarm Scoping Report. ScottishPower Renewables.
**306**	Searle, K., D. Mobbs, A. Butler, M. Bogdanova, S. Freeman, S. Wanless, and F. Daunt. 2014. Population Consequences of Displacement from Proposed Offshore Wind Energy Developments for Seabirds Breeding at Scottish SPAs. Marine Scotland Science.
**307**	Searle, K., J. Waggitt, P. Evans, M. Bogdanova, F. Daunt, and A. Butler. 2022. Study to examine the impact of climate change on seabird species off the east coast of Scotland and potential implications for environmental assessments. Centre for Ecology & Hydrology (CEH).
**308**	Serpetti, N., S. Benjamins, S. Brain, M. Collu, J. B. Harvey, J. J. Heymans, D. A. Hughes, D. Risch, S. Rosinski, J. J. Waggitt, and B. Wilson. 2021. Modeling Small Scale Impacts of Multi-Purpose Platforms: An Ecosystem Approach. Frontiers in Marine Science 8.
**309**	Shreeve, A., and G. Graeme. 2012. Rampion OWF Round 3 Zone 6: Migratory Bird Report. Natural Power.
**310**	Skov, H., M. Desholm, S. Heinänen, A. J. Kahlert, B. Laubek, E. N. Jensen, R. Žydelis, and P. B. Jensen. 2016. Patterns of migrating soaring migrants indicate attraction to marine wind farms. Biology Letters 12:20160804.
**311**	Skov, H., J. Durinck, A. Erichsen, R. Kloster, F. Møhlenberg, and S. Leonhard. 2008. Horns Rev II Offshore Wind Farm Food Basis for Common Scoter. Danish Hydraulic Institute (DHI).
**312**	Skov, H., S. Heinänen, S. Lohier, C. Thaxter, and R. Zydelis. 2012. Modelling the abundance and area use of wintering Red-throated Divers in the Outer Thames Estuary. British Trust for Ornithology (BTO).

**313**	Skov, H., S. Heinänen, T. Norman, R. Ward, S. Méndez-Roldán, and I. Ellis. 2018. ORJIP Bird Collision and Avoidance Study. Offshore Renewables Joint Industry Programme (ORJIP).
**314**	Skov, H., N. Jensen, J. Durinck, B. Jensen, and S. Leonhard. 2009. Horns Rev II Offshore Wind Farm Monitoring of Bird Migration. Danish Hydraulic Institute (DHI).
**315**	Skov, H., S. Leonhard, S. Heinänen, R. Zydelis, N. Jensen, J. Durinck, T. Johansen, B. Jensen, B. Hansen, W. Piper, and P. Grøn. 2012. Horns Rev 2 Offshore Wind Farm Bird Monitoring Program 2010-2012. Danish Hydraulic Institute (DHI).
**316**	Skov, H., W. Piper, and S. Leonhard. 2008. Horns Rev II Offshore Wind Farm Monitoring of Resting Waterbirds. Danish Hydraulic Institute (DHI).
**317**	Smetzer, J. 2018. TRACKING MIGRATORY BIRD MOVEMENTS IN THE GULF OF MAINE WITH AUTOMATED RADIO TELEMETRY AND STABLE HYDROGEN ISOTOPE MARKERS. University of Massachusetts.
**318**	Soanes, L. M., P. W. Atkinson, R. D. Gauvain, and J. A. Green. 2013. Individual consistency in the foraging behaviour of Northern Gannets: Implications for interactions with offshore renewable energy developments. Marine Policy 38:507-514.
**319**	Sovacool, K. B. 2009. Contextualizing avian mortality: A preliminary appraisal of bird and bat fatalities from wind, fossil-fuel, and nuclear electricity. Energy Policy 37:2241-2248.
**~320**	Stantec, C. 2018. South Fork Wind Farm COP Appendix Q: Avian and Bat Risk Assessment. Stantec Consulting.
**~321**	Stantec, C. 2022. Sunrise Wind Construction and Operations Plan. Stantec Consulting.
**~322**	Stantec, C. 2022. Sunrise Wind COP Appendix P1: Avian and Bat Risk Assessment. Bureau of Ocean Energy Management (BOEM).
**323**	State of, M. 2021. State of Maine Offshore Wind Energy Research Array: Application for an Outer Continental Shelf Renewable Energy Research Lease. State of Maine: Governor's Energy Office.
**324**	Stenhouse, J. I., M. A. Berlin, T. A. Gilbert, W. M. Goodale, E. C. Gray, A. W. Montevecchi, L. Savoy, and S. C. Spiegel. 2020. Assessing the exposure of three diving bird species to offshore wind areas on the U.S. Atlantic Outer Continental Shelf using satellite telemetry. Diversity and Distributions 26:1703-1714.
**325**	Stucker, J., J. Carlisle, D. Pham, and W. Erickson. 2018. Icebreaker Wind Aerial Survey Waterbird Report. Western Ecosystems Technology Inc (WEST), Cleveland, Ohio.
**326**	Sullivan, K. 2024. Atlantic Shores Offshore Wind South Final Environmental Impact Statement (FEIS) for Commercial Wind Lease OCS-A 0499. US Department of the Interior (DOI).
**327**	Suryan, R., E. Phillips, K. So, J. Zamon, R. Lowe, and S. Stephensen. 2012. Marine bird colony and at-sea distributions along the Oregon coast: Implications for marine spatial planning and information gap analysis. Oregon State University.
**~328**	Svedlow, A., L. Gilpatrick, and D. McIlvain. 2012. Appendix K Tetra Tech Bird Survey Report: Spring - Fall 2010 Avian and Bat Studies Report Lake Erie Wind Power Study. Tetra Tech Inc.
**329**	Swann, B. 2016. Seabird Counts at East Caithness Cliffs SPA for Marine Renewable Casework. Scottish Natural Heritage.

**330**	Swann, B. 2018. Seabird Counts at North Caithness Cliffs SPA in 2015 and 2016 for Marine Renewable Casework. Scottish Natural Heritage.
**331**	Swann, B. 2018. Seabird counts at St Abb's Head to Fast Castle SPA in 2016 for Marine Renewables Casework. Scottish Natural Heritage.
**332**	Tetra Tech, I. 2012. Pre-Construction Avian and Bat Survey Report. DeTect Inc.
**~333**	Tetra Tech, I. 2022. Kitty Hawk North Wind Construction and Operations Plan. Bureau of Ocean Energy Management (BOEM).
**334**	Thaxter, B. C., W. M. R. Green, P. M. Collier, C. R. Taylor, P. R. Middelveld, S. E. Scragg, J. L. Wright, P. C. S. A. Cook, and C. R. Fijn. 2024. Behavioural responses of Sandwich terns following the construction of offshore wind farms. Marine Biology 171.
**335**	Thaxter, B. C., H. V. Ross-Smith, W. Bouten, A. N. Clark, J. G. Conway, M. M. Rehfisch, and H. K. N. Burton. 2015. Seabird–wind farm interactions during the breeding season vary within and between years: A case study of lesser black-backed gull Larus fuscus in the UK. Biological Conservation 186:347-358.
**336**	Thaxter, C., D. Johnston, G. Clewley, E. Humphreys, and A. Cook. 2022. Improving Our Understanding of Seabird Behaviour at Sea. Marine Scotland Science.
**337**	Thaxter, C., V. Ross-Smith, W. Bouten, E. Masden, N. Clark, G. Conway, L. Barber, G. Clewley, and N. Burton. 2018. Dodging the blades: new insights into three-dimensional space use of offshore wind farms by lesser black-backed gulls Larus fuscus. Marine Ecology Progress Series 587:247-253.
**338**	Thaxter, C., V. Ross-Smith, N. Clark, G. Conway, A. Johnston, H. Wade, E. Masden, W. Bouten, and N. Burton. 2014. Measuring the Interaction Between Marine Features of Special Protection Areas with Offshore Wind Farm Development Sites Through Telemetry: Final Report. British Trust for Ornithology (BTO).
**339**	Thornton, P. 2014. Dogger Bank Environmental Statement - Chapter 11: Marine and Coastal Ornithology. Forewind.
**&340**	Tjørnløv, R., H. Skov, M. Armitage, M. Barker, F. Cuttat, and K. Thomas. 2021. Resolving Key Uncertainties of Seabird Flight and Avoidance Behaviours at Offshore Wind Farms: Annual report for April 2020 – October 2020. RPS group.
**&341**	Tjørnløv, R., H. Skov, M. Armitage, M. Barker, J. Jørgensen, L. Mortensen, K. Thomas, and T. Uhrenholdt. 2023. Resolving Key Uncertainties of Seabird Flight and Avoidance Behaviours at Offshore Wind Farms: Final Report for the study period 2020-2021. Danish Hydraulic Institute (DHI).
**342**	Tongue, A. 2014. Implications for Offshore Wind Farm Ornithological Cumulative Impact Assessment: How the Manipulation of Density and Breeding Season Parameters Affects the Outcomes of Collision Risk Modelling. Master's Thesis. Open University.
**343**	Tulp, I., H. Schekkerman, J. Larsen, J. van der Winden, R. van de Haterd, P. van Horssen, S. Dirksen, and A. Spaans. 1999. Nocturnal flight activity of sea ducks near the windfarm Tunø Knob in the Kattegat. Bureau Waardenburg bv.
**344**	University of, H. 2007. Seabird Survey Programme Findings, Humber Gateway Windfarm. University of Hull.

**345**	Vallejo, C. G., K. Grellier, J. E. Nelson, M. R. Mcgregor, J. S. Canning, M. F. Caryl, and N. Mclean. 2017. Responses of two marine top predators to an offshore wind farm. Ecology and Evolution 7:8698-8708.
**&346**	van der Wal, J., R. Fijn, A. Gyimesi, and M. Scholl. 2015. 2nd Iteration: Effect of turbine capacity on collision numbers for three large gull species, based on revised density data, when assessing cumulative effects of offshore wind farms on birds in the southern North Sea. IMARES - Wageningen UR.
**347**	van Kooten, T., F. Soudijn, I. Tulp, C. Chen, D. Benden, and M. Leopold. 2019. The consequences of seabird habitat loss from offshore wind turbines, version 2. Wageningen University and Research Centre.
**~348**	Vanasse Hangen, B. 2020. Revolution Wind COP Appendix K: Onshore Natural Resources and Biological Assessment. Vanasse Hangen Brustlin (VHB), Providence, RI, USA.
**349**	Vanermen, N., W. Courtens, R. Daelemans, L. Lens, W. Müller, D. V. M. Walle, H. Verstraete, and M. W. E. Stienen. 2020. Attracted to the outside: a meso-scale response pattern of lesser black-backed gulls at an offshore wind farm revealed by GPS telemetry. ICES Journal of Marine Science 77:701-710.
**350**	Vanermen, N., T. Onkelinx, W. Courtens, D. V. M. Walle, H. Verstraete, and M. W. E. Stienen. 2015. Seabird avoidance and attraction at an offshore wind farm in the Belgian part of the North Sea. Hydrobiologia 756:51-61.
**351**	Vanermen, N., T. Onkelinx, P. Verschelde, W. Courtens, D. V. M. Walle, H. Verstraete, and M. W. E. Stienen. 2015. Assessing seabird displacement at offshore wind farms: power ranges of a monitoring and data handling protocol. Hydrobiologia 756:155-167.
**352**	Vanermen, N., E. Stienen, W. Courtens, T. Onkelinx, M. Van de walle, and H. Verstraete. 2013. Bird Monitoring at Offshore Wind Farms in the Belgian Part of the North Sea - Assessing Seabird Displacement Effects. Research Institute for Nature and Forest (INBO).
**353**	Vanermen, N., E. Stienen, T. Onkelinx, W. Courtens, M. Van de walle, P. Verschelde, and H. Verstraete. 2012. Seabirds & Offshore Wind Farms Monitoring Results 2011. Research Institute for Nature and Forest (INBO).
**354**	Wade, H. M., E. A. Masden, A. C. Jackson, C. B. Thaxter, N. H. K. Burton, W. Bouten, and R. W. Furness. 2014. Great skua (Stercorarius skua) movements at sea in relation to marine renewable energy developments. Marine Environmental Research 101:69-80.
**355**	Wakefield, D. E., E. Owen, J. Baer, J. M. Carroll, F. Daunt, G. S. Dodd, A. J. Green, T. Guilford, A. R. Mavor, I. P. Miller, A. M. Newell, F. S. Newton, S. G. Robertson, A. Shoji, M. L. Soanes, C. S. Votier, S. Wanless, and M. Bolton. 2017. Breeding density, fine-scale tracking, and large-scale modeling reveal the regional distribution of four seabird species. Ecological Applications 27:2074-2091.
**356**	Walls, R., M. Brown, R. Budgey, M. Parnell, and L. Thorpe. 2004. The remote monitoring of offshore avian movement using bird detection radar at Spurn Head, East Yorkshire. Central Science Laboratory.
**357**	Walls, R., S. Canning, G. Lye, L. Givens, C. Garrett, and J. Lancaster. 2013. Analysis of Marine Environmental Monitoring Plan Data from the Robin Rigg Offshore Wind Farm, Scotland (Operational Year 1). E.ON.

**358**	Walls, R., S. Shenton, E. Knott, J. Lancaster, S. Canning, G. Lye, and C. Pendlebury. 2013. Best Practice Ecological Analysis Methods for Offshore Wind in UK: Case study: Robin Rigg Offshore Wind Farm – An integrated approach the Marine Environmental Monitoring Plan (MEMP).in 2nd Conference on Wind Energy and Wildlife Impacts (CWW 2013), Stockholm, Sweden.
**359**	Warwick-Evans, V., W. P. Atkinson, I. Walkington, and A. J. Green. 2018. Predicting the impacts of wind farms on seabirds: An individual-based model. Journal of Applied Ecology 55:503-515.
**360**	Weiser, L. E., T. C. Overton, C. D. Douglas, L. M. Casazza, and L. P. Flint. 2024. Geese migrating over the Pacific Ocean select altitudes coinciding with offshore wind turbine blades. Journal of Applied Ecology 61:951-962.
**361**	Welcker, J., and G. Nehls. 2016. Displacement of seabirds by an offshore wind farm in the North Sea. Marine Ecology Progress Series 554:173-182.
**362**	Wells, R., and S. Shenton. 2011. Robin Rigg Offshore Wind Farm Marine Environmental Monitoring Plan (MEMP): Ecological Analysis Data. Page 44.
**363**	White, C., Y.-H. Wang, K. R. Walter, I. B. Ruttenberg, D. Han, E. Newman, R. E. Deyle, S. Gopal, and L. Kaufman. 2024. Spatial planning offshore wind energy farms in California for mediating fisheries and wildlife conservation impacts. Environmental Development 51:101005.
**364**	Williams, K. 2013. Modeling Wildlife Densities and Habitat Use Across Temporal and Spatial Scales on the Mid-Atlantic Continental Shelf: Annual Report for the First Budget Period. Biodiversity Research Institute.
**365**	Willmot, J., G. Forcey, M. Vukovich, S. McGovern, J. Clerc, and J. Carter. 2021. Ecological Baseline Studies of the US Outer Continental Shelf. National Institute for Environmental Studies (NIES), Bedford, NH.
**366**	Willmott, J., G. Forcey, and A. Kent. 2013. The Relative Vulnerability of Migratory Bird Species to Offshore Wind Energy Projects on the Atlantic Outer Continental Shelf: An Assessment Method and Database. Normandeau Associates Inc.
**367**	Willmott, R. J., G. Forcey, and M. Vukovich. 2023. New insights into the influence of turbines on the behaviour of migrant birds: implications for predicting impacts of offshore wind developments on wildlife. Page 012006 Journal of Physics: Conference Series.
**368**	Wind, S. M. 2012. Hornsea Project 2 Environmental Impact Assessment. SMart Wind.
**369**	Winiarski, J. K., L. D. Miller, W. C. P. Paton, and R. S. Mcwilliams. 2014. A spatial conservation prioritization approach for protecting marine birds given proposed offshore wind energy development. Biological Conservation 169:79-88.
**370**	Winiarski, K., D. Miller, P. Paton, and S. Mcwilliams. 2013. Spatially explicit model of wintering common loons: conservation implications. Marine Ecology Progress Series 492:273-283.
**371**	Wisman, J., and S. Maxwell. 2018. Potential Interaction Analysis of Offshore Wind Energy Areas and Breeding Avian Species on the US Mid-Atlantic Coast. Master's Thesis. Old Dominion University.

**372**	Woodward, I., S. Franks, K. Bowgen, J. Davies, R. Green, L. Griffin, C. Mitchell, N. O'Hanlon, C. Pollock, E. Rees, C. Tremlett, L. Wright, and A. Cook. 2023. Strategic study of collision risk for birds on migration and further development of the stochastic collision risk modelling tool. Scottish Government.
**373**	Wright, L., V. Ross-Smith, G. Austin, D. Massimino, D. Dadam, A. Cook, N. Calbrade, and N. Burton. 2012. Assessing the risk of offshore wind farm development to migratory birds designated as features of UK Special Protection Areas (and other Annex 1 species). British Trust for Ornithology (BTO).
**374**	(WWT), W. a. W. T. 2014. Strategic assessment of collision risk of Scottish offshore wind farms to migrating birds. Scottish Marine and Freshwater Science 5:174.
**375**	Xodus, G. 2015. Hywind Scotland Pilot Park Seabirds and Marine Mammals Technical Report. Xodus Group.
**376**	Zipkin, F. E., P. B. Kinlan, A. Sussman, D. Rypkema, M. Wimer, and F. A. O'Connell. 2015. Statistical guidelines for assessing marine avian hotspots and coldspots: A case study on wind energy development in the U.S. Atlantic Ocean. Biological Conservation 191:216-223.
