# Supplementary material for: Mapping the Evidence for Cumulative Adverse Effects of Offshore Wind Installations on Birds
Source: Environ Manage. 2026 Aug 21;76(9):283. doi: 10.1007/s00267-026-02599-7 (PMC13498612; doi:10.1007/s00267-026-02599-7)
Supplement: Supplementary file 4 [file 267_2026_2599_MOESM4_ESM.pdf]

Supplementary Table 2. Table detailing the explicit inclusion criteria used for each category in our systematic review.

Category	Criteria
**Source - OSW (SHWF)**	• Multiple OSW farms included<br>• Analysis or summary statistic of cumulative effect/exposure<br>• Not just an overlay of presence or density
**Source - non-OSW (SHO)**	• Analysis of hazards other than OSW farms (e.g. fisheries, shipping, natural resource extraction, etc.)
**Effect Pathways (EP)**	• Analysis of how multiple OSW farms collectively impact birds.<br>• Impact can be additive, multiplicative (synergistic), or inhibitory (antagonistic)<br>• Must analyze the nature of the effect pathway, cannot just assume one
**Refine Receptors (RR)**	• Data for, or analysis of, a species that helps determine the risk posed by OSW<br>• Must be species specific<br>• Includes sensitivity indices, flight height measurements, and calculated avoidance rates
**Clear Baselines (CB)**	• Data collected for a species prior to the construction of offshore wind farm(s)<br>• Must be species specific<br>• Includes population estimates, density maps, distribution maps, and tracking data in and around the proposed OSW site
**Stating a Threshold (ST)**	• Sets a level of acceptable mortality given the species biology<br>• Must be species specific<br>• Includes calculating potential biological removal
**Temporal - Lifespan (TB1)**	• Impacts of OSW on birds throughout the full life cycle of the OSW facility (construction, operations, dismantling)<br>• Must do some level of analysis with species from the region of interest (can not be a summary of potential effects on birds generally)
**Temporal - Future Anthropogenic (TB2)**	• Analysis of future anthropogenic actions that can influence cumulative impacts<br>• Any impact from SHWF and SHO above, if that impact is anticipated for the future<br>• Must have some relation to current risks from OSW<br>• Any analysis of the impact of climate change on birds in the context of OSW

Category	Criteria
**Temporal - Life History (TB3)**	• Data on a species' annual cycle and how that influences exposure or risk to OSW<br>• Must be species specific<br>• Can be year-round data or data from a season of interest (e.g. variation in behavior/abundance throughout breeding season)<br>• Generally takes the form of abundance/frequency data
**Spatial - Population (SBP)**	• Mapping of species distribution, occupancy, habitat suitability, or telemetry tracks<br>• Can be mapped throughout annual cycle or for a subset of the cycle (e.g. breeding season)<br>• Telemetry data without arbitrary geographical cutoffs always qualifies<br>• Maps based on species distribution models or surveys other than telemetry must be of a sufficient size to encapsulate the population<br>• Maps at the level of a country or state/province (in the United States/Canada/Australia) qualify<br>• Maps of the area surrounding a breeding colony that are based on the species biology also qualify
**Spatial - Development Area (SBD)**	• Marine spatial planning that incorporate bird biology/ecology in determining optimal siting locations for OSW farms<br>• Does not need to be species specific
